# Marine Natural Peptides: Determination of Absolute Configuration Using Liquid Chromatography Methods and Evaluation of Bioactivities

**DOI:** 10.3390/molecules23020306

**Published:** 2018-01-31

**Authors:** Ye’ Zaw Phyo, João Ribeiro, Carla Fernandes, Anake Kijjoa, Madalena M. M. Pinto

**Affiliations:** 1 ICBAS-Instituto de Ciências Biomédicas Abel Salazar, Universidade do Porto, Rua de Jorge Viterbo Ferreira, 228, 4050-313 Porto, Portugal; chemistkophyo.ckp@gmail.com; 2 Interdisciplinary Centre of Marine and Environmental Research (CIIMAR), Edifício do Terminal de Cruzeiros do Porto de Leixões, Av. General Norton de Matos s/n, 4050-208 Matosinhos, Portugal; madalena@ff.up.pt; 3 Laboratório de Química Orgânica e Farmacêutica, Departamento de Ciências Químicas, Faculdade de Farmácia da Universidade do Porto, Rua de Jorge Viterbo Ferreira, 228, 4050-313 Porto, Portugal; joaobigi@gmail.com

**Keywords:** absolute configuration, bioactivity, chiral HPLC, Marfey’s method, marine peptides, stereochemistry

## Abstract

Over the last decades, many naturally occurring peptides have attracted the attention of medicinal chemists due to their promising applicability as pharmaceuticals or as models for drugs used in therapeutics. Marine peptides are chiral molecules comprising different amino acid residues. Therefore, it is essential to establish the configuration of the stereogenic carbon of their amino acid constituents for a total characterization and further synthesis to obtain higher amount of the bioactive marine peptides or as a basis for structural modifications for more potent derivatives. Moreover, it is also a crucial issue taking into account the mechanisms of molecular recognition and the influence of molecular three-dimensionality in this process. In this review, a literature survey covering the report on the determination of absolute configuration of the amino acid residues of diverse marine peptides by chromatographic methodologies is presented. A brief summary of their biological activities was also included emphasizing to the most promising marine peptides. A case study describing an experience of our group was also included.

## 1. Introduction

In recent years, it has become well known that the oceans represent a rich source of structurally unique bioactive compounds from the perspective of potential therapeutic agents [1,2]. Bioactive compounds can be isolated from a myriad of marine invertebrates such as mollusks, sponges, tunicates and bryozoans, in addition to algae and marine microorganisms, especially cyanobacteria, bacteria and fungi [3,4,5].

Over the last decades, novel bioactive compounds from marine organisms with important bioactivities, such as antifungal, antibacterial, cytotoxic and anti-inflammatory properties, have been widely explored, and many of them are considered as lead compounds for drug discovery as well as biologically useful agents in pharmaceutical research [6,7,8,9,10]. In fact, owing to their pharmacological potential, either directly as drugs or as models for molecular modifications and/or total synthesis, marine natural products are certainly an interesting source, exploited by many researchers [11].

Ziconotide (Prialt^®^), a peptide first isolated from the venom of the cone snail (*Conus magus*), and trabectedin (Yondelis^®^), an alkaloid originally isolated from a marine tunicate *Ectenascidia turbinata* and now obtained by semisynthesis, are examples of marine natural products that have already been approved as human therapeutics [3,12,13,14]. Ziconotide is an analgesic used for treatment of patients suffering from chronic pain, and trabectedin for the treatment of soft tissue sarcomas and ovarian cancer.

In terms of the overall number of marine natural products, peptides are one of the most described due to their novel chemistry and diverse biological properties [15]. Actually, marine peptides are known to exhibit various biological activities such as antiviral, antiproliferative, antioxidant, anticancer, antidiabetic, anti-obesity, anticoagulant, antihypertensive, and calcium-binding activities [6,15,16,17].

Marine peptides are chiral molecules comprising different amino acid residue subunits. For their total characterization, and taking into account the mechanisms of molecular recognition and the influence of molecular three-dimensionality in this process, it is essential to define the configuration of the amino acids components of the peptide fractions, isolated from marine sources. Besides, it is also crucial to obtain the bioactive marine peptides by synthesis in order to achieve higher amount of compound for future assays or as a basis for structural modifications to obtain more potent derivatives.

Nowadays, there are different methodologies for the determination of the absolute configuration of amino acids, such as X-ray crystallography, NMR techniques, vibrational circular dichroism (VCD), enantioselective chromatography, optical rotatory dispersion (ORD), among others [18,19,20,21,22,23,24,25,26].

**Figure 1:**
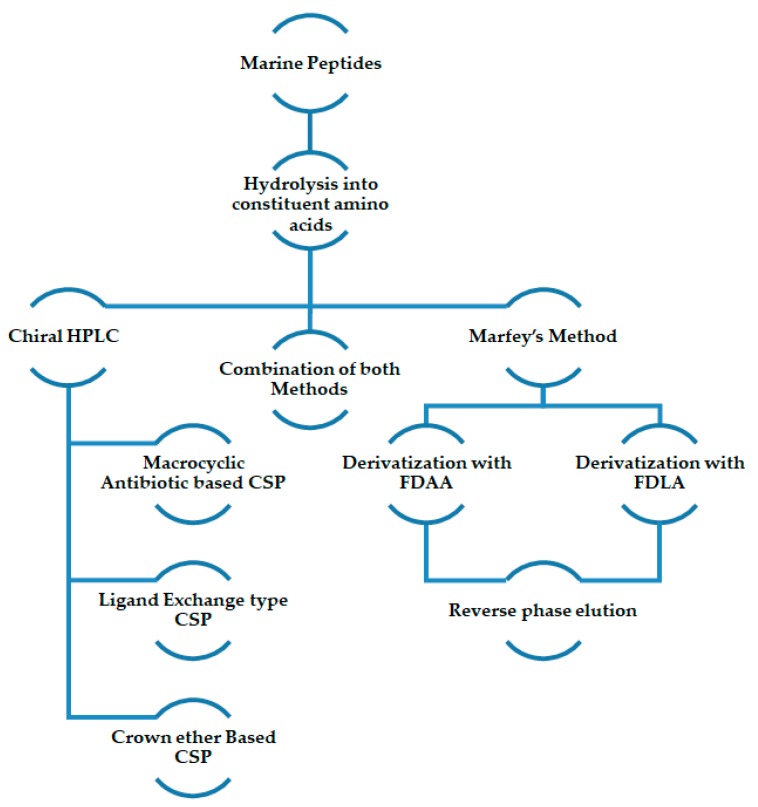
Schematic presentation of the methodologies generally used for determination of the configuration of amino acid residues of marine peptides. HPLC—High Performance Liquid Chromatography; CSP—Chiral Stationary Phase; FDAA—1-Fluoro-2-4-dinitrophenyl-5-d,l-alanine amide; FDLA—1-Fluoro-2-4-dinitrophenyl-5-d,l-leucine amide.

For the determination of the absolute configuration of amino acid residues of marine peptides, separation methodologies by using Marfey’s method, chiral high performance liquid chromatography (HPLC) analysis or both have proved to be suitable and the most described, as will be shown in this review. Regardless of the method used, the evaluation of peptides stereochemistry is based on the determination of the amino acid composition in peptide hydrolysates. Two main steps are involved, specifically the total or partial hydrolysis of peptides to obtain amino acid residues followed by their analysis by comparison with appropriated standards [27] (Figure 1).

Marfey’s method was first reported by Marfey in 1984 [28]. After the acid hydrolysis of peptides, the amino acid residues are derivatized with chiral Marfey’s reagents such as 1-fluoro-2-4-dinitrophenyl-5-d,l-alanine amide (FDAA) or 1-fluoro-2-4-dinitrophenyl-5-d,l-leucine amide (FDLA). Subsequent analysis via reverse phase liquid chromatography (LC), using generally C_18_ columns, and by comparison the retention times of the derivatized amino acids with suitable standards, afforded the stereochemistry of the peptides [29,30,31]. This method is often used for determination of the absolute configuration of amino acids, mainly because it is a simple method, offering a better resolution when compared to chiral HPLC methodologies; furthermore, several derivatization agents, such as FDAA and FDLA, are commercially available. However, this methodology has some disadvantages, including low availability of some standards, and the possibility of occurring racemization of the analyte during the derivatization reaction, prior to the chromatographic analysis [30,31].

The chiral analysis by HPLC is based on a formation of transient diastereomeric complexes between the amino acids present in the hydrolysates and the chiral stationary phase (CSP) employed, being the less stable complex the first to elute [32]. There are several types of CSPs, such as polysaccharide-based, Pirkle-type, protein-based, macrocyclic antibiotic-based, crown ether-based, ligand exchange type, among others [33,34,35]; however, the last three types are the most used for the separation of primary amine-containing compounds and amino acids [36,37]. Chiral HPLC offers several advantages, when comparing to Marfey’s method, including the direct analysis of the amino acid hydrolysates without further derivatization; moreover, the analysis often provides quicker results. However, poor chemical sensitivity, low sample capacity, and low availability and expensiveness of commercial chiral columns are some of the disadvantages of chiral HPLC method [38].

A number of reviews on marine peptides have appeared in recent years, focusing mainly on their biological activities, applications and biosynthesis as well as isolation procedures [16,39,40,41,42,43,44,45,46,47,48,49,50,51,52,53,54,55,56,57]. In this review, several works related to the methods used for determination of the absolute configuration of marine peptides by chromatographic methods are presented in different sections according to the source of the marine peptides. Diverse types of peptides such as cyclic peptides, cyclic depsipeptides and lipopeptides are reported. A literature survey covering all the reports on liquid chromatographic methods (Marfey’s method and chiral HPLC) is presented (from 1996 to 2017). Furthermore, a case study describing an experience of our group is included.

## 2. Peptides from Marine Cyanobacteria and Other Bacteria

Cyanobacteria (blue-green algae), the most ancient known microorganisms on Earth, are a rich source of novel secondary metabolites possessing a broad spectrum of biological activities including antitumor, antibacterial, anticoagulant, antifungal, antiviral, antimalarial, antiprotozoal, and anti-inflammatory activities [58]. Currently, cyanobacteria are one of the most interesting sources of novel marine compounds [59]. Actually, the number of biologically active cyclic peptides, depsipeptides, lipopeptides, and other acyclic or small peptides, many of which containing unusual amino acid residues or modified amino acid units, is impressive. In addition to cyanobacteria, this type of compounds has also been isolated from other marine-derived bacteria.

### 2.1. Cyclic Peptides

**Figure 2:**
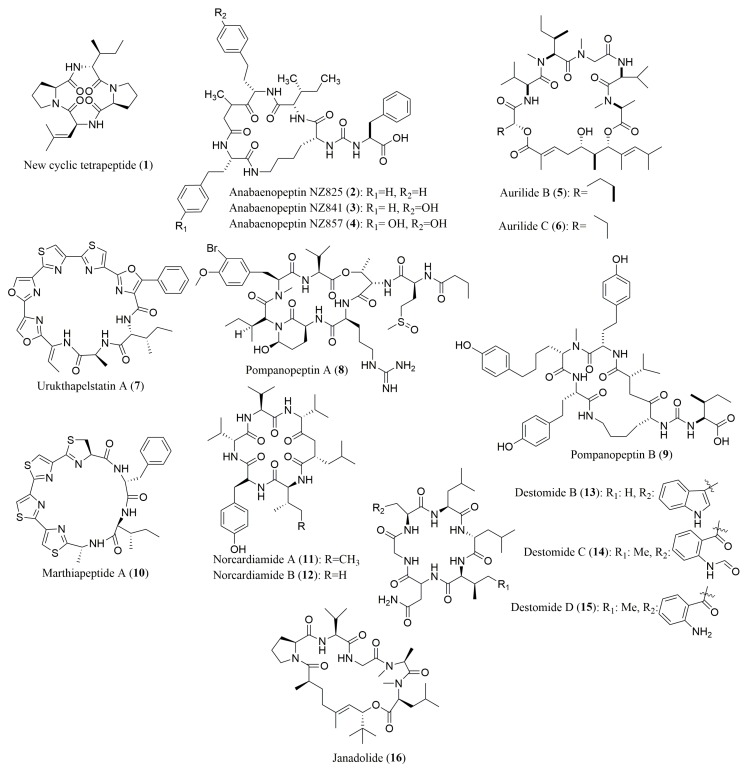
Structure of cyclic peptides **1**–**16**, isolated from marine cyanobacteria and other bacteria, whose stereochemistry determination of their amino acids was performed by Marfey’s method (compounds **1**–**4**) and by a combination of both Marfey’s method and chiral HPLC (compounds **5**–**16**).

Scattered publications concerning the stereochemistry determination of the amino acid residues of several cyclic peptides, isolated from marine cyanobacteria and other bacteria, were reported (Table 1). Marfey’s method, using FDAA as derivatization reagent, allowed the successful determination of the absolute configuration of the amino acid residues of cyclic peptides **1**–**4** (Figure 2).

For the new cyclic tetrapeptide **1** isolated from the bacterium *Nocardiopsis* sp. [60], the absolute configuration of all the amino acid residues was found to be L. Similarly, the absolute configuration of the amino acid residues of three novel anabaenopeptins labeled NZ825 (**2**), NZ841 (**3**), and NZ857 (**4**) [61], were successfully determined by Marfey’s method combined with HPLC.

However, as Marfey’s method was not accurate enough to determine the absolute configuration of all the amino acid residues of some cyclic peptides **5**–**16** (Figure 2), it was necessary to associate this method with chiral HPLC.

This strategy, i.e., using a ligand exchange-type CSP in chiral HPLC associated with Marfey’s method, was used for the determination of amino acids stereochemistry of several cyclic peptides, including aurilide B (**5**) and C (**6**), isolated from the cyanobacterium *Lyngbya majuscula* [62], urukthapelstatin A (**7**), isolated from a culture broth of thermoactinomycetaceae bacterium *Mechercharimyces asporophorigenens* YM11-542 [63], pompanoeptpins A (**8**) and B (**9**), isolated from the cyanobacterium *Lyngbya confervoides* [64], marthiapeptide A (**10**) isolated from the deep South China Sea-derived *Marinactinospora thermotolerance* SCSIO 00652 [65], norcardiamides A (**11**) and B (**12**), isolated from the marine-derived actinomycete *Nocardiopsis* sp. CNX037 [66], destomides B–D (**13**–**15**), isolated from the deep South China Sea-derived *Streptomyces scopuliridis* SCSIO ZJ46 [67], and jandolide (**16**) isolated from the marine cyanobacterium *Okeania* sp. [68].

The cyclic peptides aurilides B (**5**) and C (**6**) were reported to have the in vitro cytotoxicity toward NCl-H460, human lung tumor, and neuro-2a mouse neuroblastoma cell lines, with lethal concentration 50 (LC50) values between 0.01 and 0.13 µM [62]. Aurilide B (**5**) was evaluated in the NCl 60 cell line panel and was found to exhibit a high level of cytotoxicity, particularly against leukemia, renal, and prostate cancer cell lines [62]. The cyclic peptide pompanopeptpin A (**8**) was shown to exhibit trypsin inhibitory activity with an IC_50_ value of 2.4 ± 0.4 µg/mL [64]. A polythiazole cyclopeptide, marthiapeptide A (**10**) showed antibacterial activity against a panel of Gram-positive bacteria with minimum inhibitory concentration (MIC) values ranging from 2.0 to 8.0 μg/mL, and strong cytotoxicity against a panel of human cancer cell lines with IC_50_ values ranging from 0.38 to 0.52 μM [65]. The cyclohexapeptide destomide B (**13**) also showed antimicrobial activity against *Staphylococcus aureus* ATCC 29213, *Streptococcus pneumoniae* NCTC 7466 and MRSE shhs-E1 with MIC values of 16.0, 12.5, 32.0 µg/mL, respectively [67]. A cyclic polyketide-peptide hybrid, janadolide (**16**) exhibited potent antitrypanosomal activity with an IC_50_ value of 47 nM [68]. 

**Figure 3:**
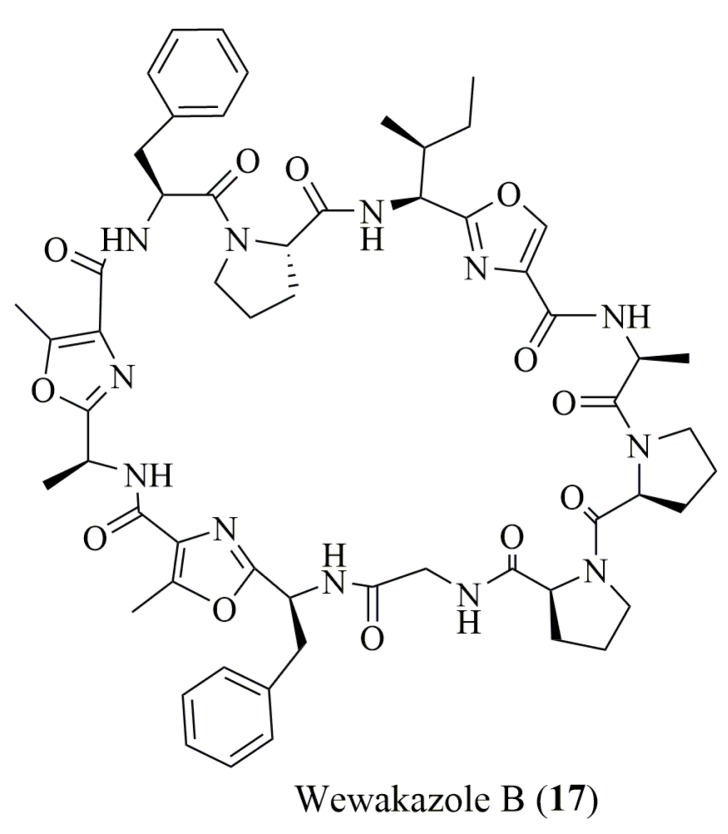
Structure of wewakazole B (**17**) isolated from a marine cyanobacteria.

Recently, the configuration of the amino acids of a cytotoxic cyanobactin, wewakazole B (**17**), isolated from the cyanobacterium *Moorea producens* (Figure 3), was determined using only chiral HPLC [69]. Two different types of CSPs, under reverse phase mode, were used to perform the analysis. A macrocyclic antibiotic-based CSP afforded the assignment of the l-configuration for its Ala, Phe, and Pro residues, while a ligand exchange type CSP clearly identified the presence of l-Ile, which could not be distinguished by the first CSP [69].

### 2.2. Cyclic Depsipeptides

**Figure 4:**
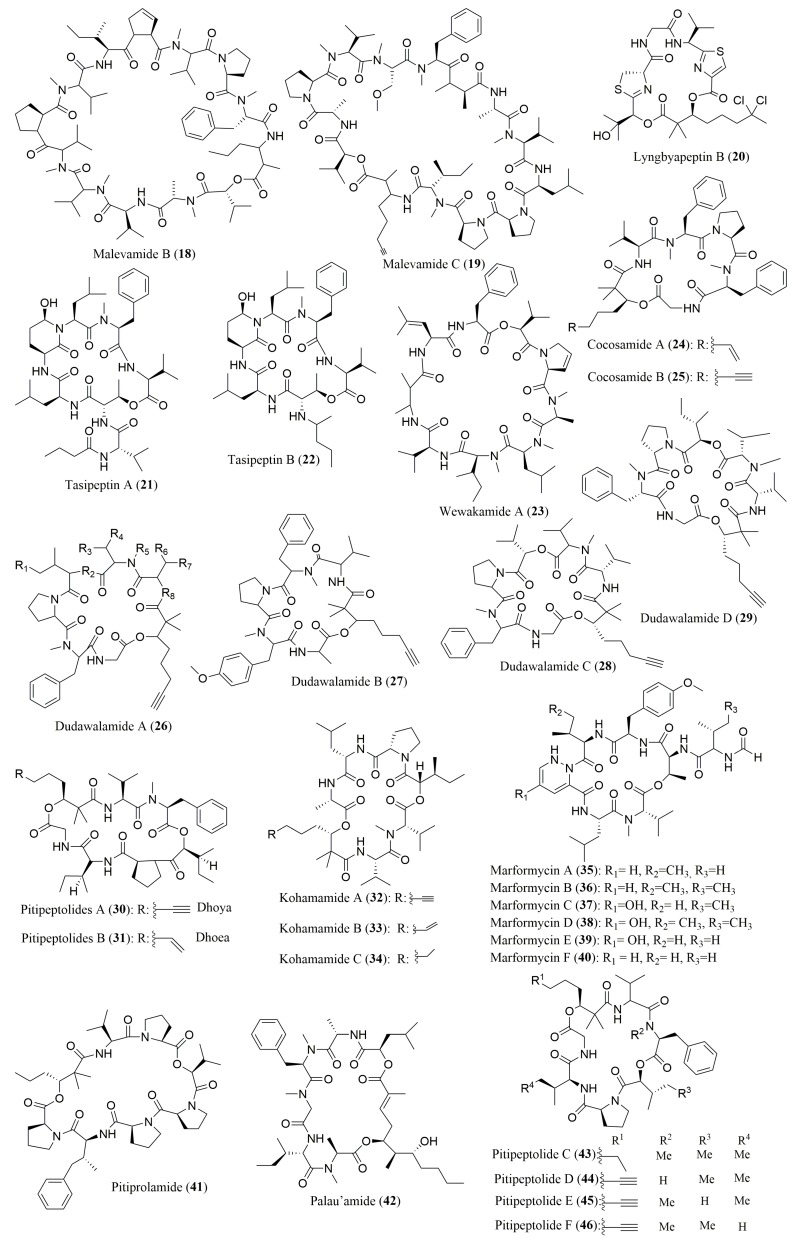
Structure of cyclic depsipeptides **18**–**46**, isolated from marine cyanobacteria and other bacteria, whose stereochemistry of their amino acids was determined only by chiral HPLC.

As mentioned above, there are many publications describing the isolation and characterization, including the determination of the stereochemistry of their amino acids, of new cyclic depsipeptides from marine cyanobacteria and other bacteria (Table 2). However, contrary to cyclic peptides, several works reported the use of chiral HPLC as the only method for determination of the configuration of amino acids. Figure 4 shows the structure of cyclic depsipeptides **18**–**46**, isolated from marine cyanobacteria and other bacteria, whose stereochemistry of the amino acids was determined only by this method.

The ligand exchange-type CSPs were the most widely used by different research groups. Cai et al. employed a penicillamine ligand exchange-type CSP to determine the absolute configuration of the amino acids constituent of malevamide B (**18**) and C (**19**) isolated from the cyanobacterium *Symploca laete-viridis* [71]. Three different mobile phases in reverse phase elution mode were used. Nevertheless, the stereochemistry of Amha and Amoa residues present in both compounds were not determined [71]. The same CSP was employed to establish that all the amino acids of the cytotoxic depsipeptide lyngbyapeptin B (**20**) [72], tasipeptins A (**21**) and B (**22**) [73], wewakamide A (**23**) [74], cocosamide A (**24**) and B (**25**) [75], and the antiparasitic depsipeptides dudawelamides A–D (**26**–**29**) [76], isolated from cyanobacteria *Lyngbya majuscula*, *Symploca* sp., *Lyngbya semiplena*, *Lyngbya majuscula*, and *Moorea producens*, respectively, has l-configuration. The only exception was for *allo*-Hiva amino acid of dudawelamide C (**29**), which has d-configuration [76]. The configuration of the amino acids of the cyclic depsipeptides pitipeptolides A (**30**) and B (**31**), isolated from cyanobacterium *Lyngbya majuscula*, was assigned to be L by a ligand exchange-type CSP comprising *N*,*N*-dioctyl-l-alanine as chiral selector (Chiralpack MA (+) from Daicel) and different proportion of CuSO_4_:ACN as mobile phase [77]. By using the same CSP, the absolute configuration of three new cyclic depsipeptides, kohamamides A–C (**32**–**34**) were also successfully established [78].

Zhou et al. [79] described the determination of the absolute configuration of new anti-infective cycloheptadepsipeptides marformycins A–F (**35**–**40**), produced by the deep sea-derived *Streptomyces drozdowiczii* SCSIO 1014, using a ligand exchange type CSP containing the same chiral selector as the previous ones (*N*,*N*-dioctyl-l(or d)-alanine) but purchased from Mitsubishi Chemical Corporation (MCI GEL CRS10W). Another type of CSP, specifically the macrocyclic antibiotic-based Chirobiotic TAG, confirmed the presence of l-Pro and l-Val in an unusual cyclic depsipeptide, pitiprolamide (**41**), isolated from *Lyngbya majuscula* [80]. Interestingly, in some works, more than one CSP were employed to elucidate the configuration of all the amino acids contained in the hydrolysates of cyclic depsipeptides. For example, two different types of ligand exchange type CSPs were used to elucidate the stereochemistry of the amino acid residues of palau’amide (**42**), depsipeptide with strong cytotoxicity against KB cell line (IC_50_ value of 13 nM) [81].

In the case of pitipeptolides C–F (**43**–**46**), which were isolated from the cyanobacterium *Lyngbya majuscula*, the configuration of most of the amino acid residues was determined using the macrocyclic antibiotic-based Chirobiotic TAG under reverse phase elution conditions [82]. Then, the *N*,*N*-dioctyl-l-alanine ligand exchange CSP Chiralpack MA (+), under the same elution mode, was used for the assignment of *S* configuration for Hiva residue [82].

**Figure 5:**
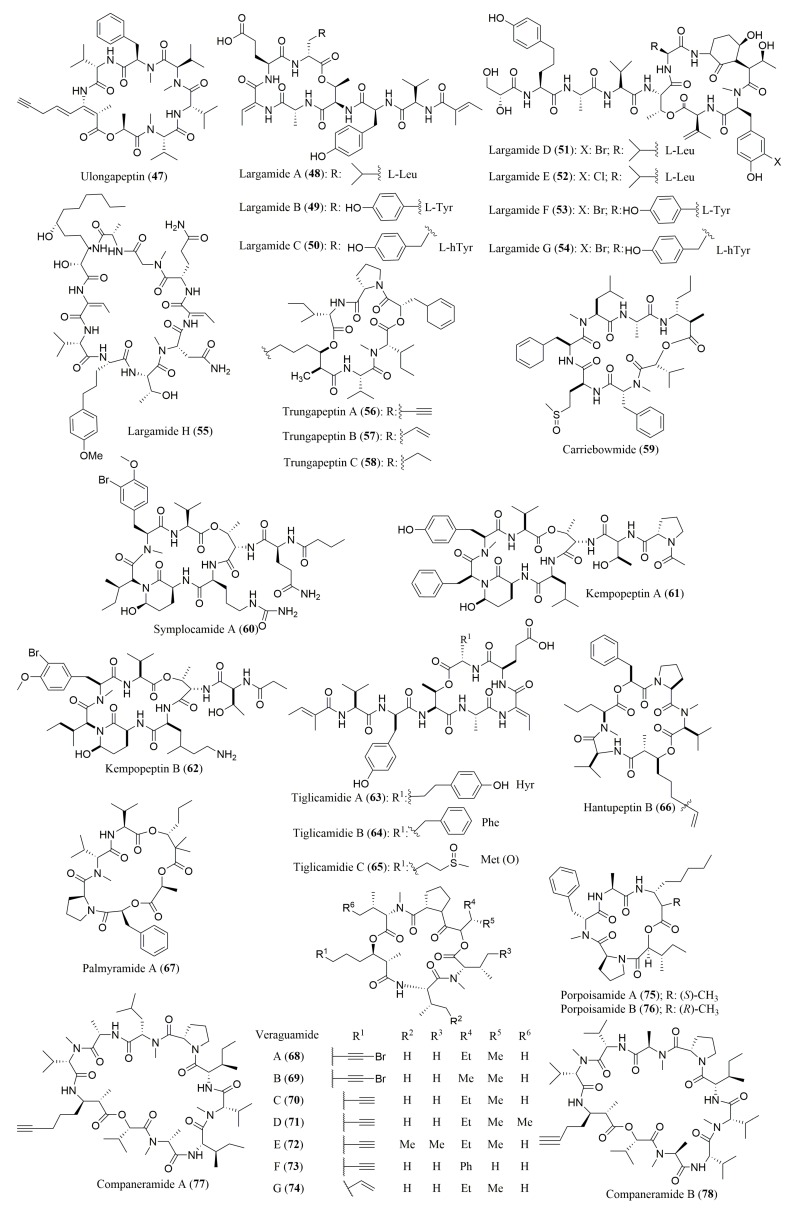
Structure of cyclic depsipeptides **47**–**78**, isolated from marine cyanobacteria and other bacteria, whose stereochemistry of their amino acids was determined by a combination of Marfey’s method and chiral HPLC.

The concurrent applicability of chiral HPLC and Marfey’s methods for determination of the absolute configuration of all the amino acid residues of cyclic depsipeptides **47**–**78** (Figure 5) was also described in several reports, among which ten described the use of ligand exchange-type CSPs to perform the analysis in association with Marfey’s method [71,72,74,75,76,78,79,81]. Furthermore, the use of macrocyclic antibiotic-based CSPs was reported by Montaser et al. [82].

Considering the biological activities of cyclic depsipeptides, whose stereochemistry of the amino acids was determined by a combination of Marfey’s method and chiral HPLC, it is worth mentioning the following compounds. Ulongapeptin (**47**), isolated from a Palauan marine cyanobacterium *Lyngbya* sp. displayed significant cytotoxic activity against KB cells with IC_50_ value of 0.63 µM [83]. Largamides A–H (**48**–**55**), isolated from the marine cyanobacterium *Oscillatoria* sp., inhibited chymotrypsin with IC_50_ values ranging from 4 to 25 µM [84]. Symplocamide A (**60**), isolated from the marine cyanobacterium *Symploca* sp., showed cytotoxicity against NCI-460, non-small cell lung cancer cells (IC_50_ = 40 nM), and neuro-2a mouse neuroblastoma cells (IC_50_ = 29 nM). It was also reported that **60** was active against three tropical parasites: malaria (*Plasmodium falciparum*, IC_50_ = 0.95 µM), chagas disease, (*Trypanasoma cruzi*, IC_50_ > 9.5 µM), and leishmaniasis (*Leishmania donovani*, IC_50_ > 9.5 µM) [87]. It was found that, kempopeptins A (**61**) and B (**62**), isolated from the marine cyanobacterium *Lyngbya* sp., exhibited inhibitory activity against elastase and chymotrypsin with IC_50_ values of 0.32 µM and 2.6 µM, respectively [88]. Palmyramide A (**67**), isolated from the marine cyanobacterium *Lyngbya majuscula*, showed sodium channel blocking activity in the neuro-2a cells as well as cytotoxic activity in H-460 human lung carcinoma cell line [91]. Companeramides A (**77**) and B (**78**), isolated from a marine cyanobacterial assemblage comprising a small filament *Leptolyngbya* species, showed high nanomolar in vitro antiplasmodial activity against *Plasmodium falciparum* strains D6, Dd2, and 7G8 [94].

**Figure 6:**
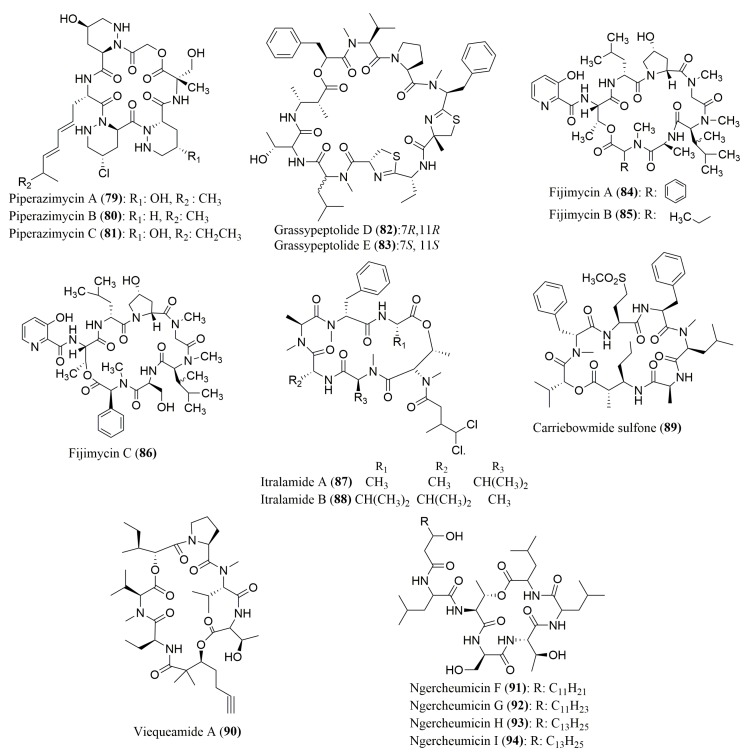
Structure of cyclic depsipeptides **79**–**94**, isolated from marine cyanobacteria and other bacteria, whose stereochemistry of their amino acids was determined by Marfey’s method.

Moreover, HPLC analysis after derivatization with a Marfey’s reagent has been reported as the only method to determine the stereochemistry of the amino acid residues of cyclic depsipeptides **79**–**94** (Figure 6). FDAA was used as derivatization reagent for piperazimycins A–C (**79**–**81**), cyclic hexadepsipeptides isolated from the fermentation broth of a marine-derived bacterium *Streptomyces* sp. Strain, collected from a sediment [95], grassypeptolides D (**82**) and E (**83**), cyclic depsipeptides isolated from the marine cyanobacterium *Leptolyngbya* sp. [96], fijimycins A–C (**84**–**86**), cyclic depsipeptides isolated from a marine bacteria *Streptomyces* sp. [97]. The Marfey’s reagent FDLA was employed for the assignment of the absolute configuration of the amino acid residues of several cyclic depsipeptides such as itralamide A (**87**) and B (**88**) and carriebowmide sulfone (**89**), isolated from the marine cyanobacterium *Lyngbya majuscula* [98], viequeamide A (**90**), isolated from the marine button cyanobacterium (*Rivularia* sp.) [99], ngercheumicins F–I (**91**–**94**) [100].

Many cyclic depsipeptides whose stereochemistry of their amino acids was determined only by Marfey’s method, exhibited various interesting biological activities. Thus, piperazimycin A (**79**) was found to exhibit potent cytotoxicity against a panel of sixty cancer cell lines (mean values of growth inhibition (GI_50_) = 100 nM, and LC_50_ = 2 µM) [95]. While, grassypeptolides D (**82**) and E (**83**) exhibited significant cytotoxicity to HeLa (IC_50_ = 335 and 192 nM, respectively) and mouse neuro-2a blastoma (IC_50_ = 559 and 407 nM, respectively) cell lines [96], itralamide B (**88**) was active against HEK293 cells (IC_50_ value of 6 ± 1 µM) [98]. Fijimycins A–C (**84**–**86**) exhibited strong growth inhibitory activity against three MRSA strains in a concentration range of 4–32 µg/mL^−1^ [97].

### 2.3. Lipopeptides

**Figure 7:**
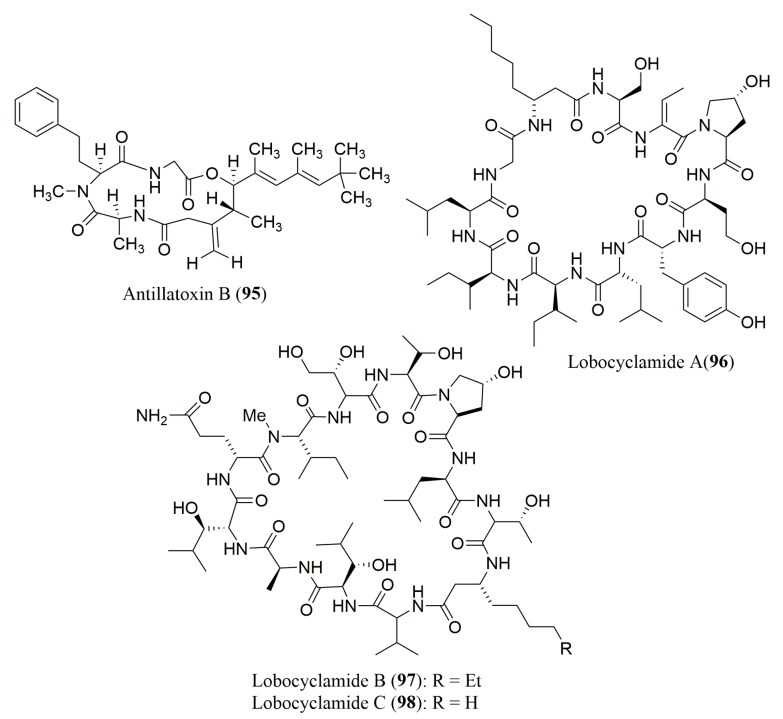
Structure of lipopeptides **95**–**98**, isolated from marine cyanobacteria.

To the best of our knowledge, there are only two reports describing simultaneously the isolation and characterization of lipopeptides from marine cyanobacteria (Figure 7) as well as the stereochemistry determination of the amino acids present in their hydrolysates (Table 3).

The configuration of *N*-Me-Hph of the lipopeptide antillatoxin B (**95**), isolated from the cyanobacterium *Lyngbya majuscula*, was assigned as L using FDAA as Marfey’s derivatization reagent [101]. Compound **95** exhibited significant sodium channel activation (EC_50_ = 1.77 μM) and ichthyotoxicity (LC_50_ = 1 μM) [101]. The hydrolysates of lipopeptides lobocyclamides A–C (**96**–**98**), isolated from the cyanobacterium *Lyngbya confervoides*, were analyzed by either direct chiral HPLC, using the d-penicillamine ligand exchange type CSP or by prior derivatization by Marfey’s method and reverse phase HPLC [102]. Both compounds displayed modest in vitro antifungal activity against a panel of *Candida* sp., including two fluconazole-resistant strains. Interestingly, synergistic antifungal activity was also observed [102].

## 3. Peptides from Marine-Derived Fungi

Marine fungi have been isolated from various marine sources like algae, marine invertebrates, sediment or water, mangroves and sponges. Most of the fungal species isolated from marine sponges are related to the genera *Aspergillus* and *Penicillium* [103]. Marine fungi are a rich source of structurally unique and biologically active compounds with a wide range of biological activities, such as antimalarial, anticancer, antifungal, antibacterial, cytotoxicity and among others [104]. More than 1000 compounds have been already isolated from marine derived fungi and among them around 150–200 new compounds were bioactive [103,104].

### 3.1. Cyclic Peptides

**Figure 8:**
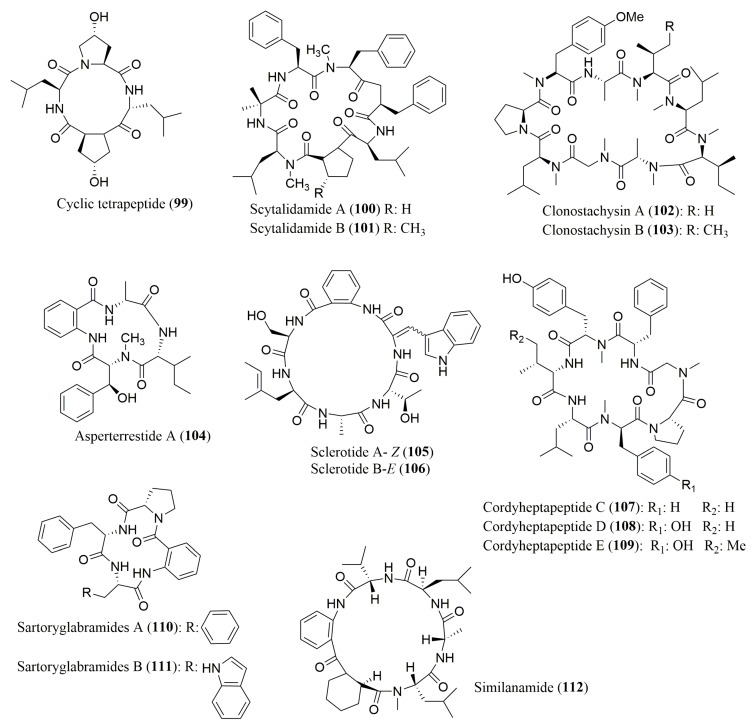
Structure of cyclic peptides **99**–**112**, isolated from marine-derived fungi.

A large number of cyclic peptides have been isolated from marine-derived fungi (Figure 8) and Table 4 shows the marine fungal cyclic peptides whose stereochemistry of their amino acid residues were determined. To the best of our knowledge, only three reports described the use of FDAA and FDLA as Marfey’s derivatization reagents, specifically for analysis of the peptides **99**–**112**.

The cyclic peptide cyclo-(l-leucyl)-*trans*-4-hydroxyl-l-prolyl-d-leucyl-*trans*-4-hydroxy-l-proline) (**99**), isolated from the marine mangrove-derived fungi *Phomopsis* sp. K38, and *Alternaria* sp. E33, was found to exhibit antifungal activity, particularly the fungus *Helminthosporium sativum*. By using a combination of Marfey’s method and a reverse phase HPLC, the presence of 4-OH-l-Pro and both l- and d-Leu residues in its structure was confirmed [105]. Scytalidamides A (**100**) and B (**101**), and clonostachysins A (**102**) and B (**103**), isolated from marine sponge derived fungus *Clonostachys rogersoniana* strain HJK9, were found to comprise l-configuration for all their amino acids [106,107]. Scytalidamides A (**100**) and B (**101**) showed cytotoxicity against human colon carcinoma tumor cell line (HCT-116) with IC_50_ values of 2.7 and 11.0 µM, respectively, and the NCI 60 cell-line, with 7.9 and 4.1 µM GI-50, respectively [106], while clonostachysins A (**102**) and B (**103**) exhibited inhibitory effect on the *Prorocentrum micans* alga at concentration higher than 30 µM [107].

Both Marfey’s method and chiral HPLC analysis were also used for the analysis of the absolute configuration of the amino acids of asperterrestide A (**104**), a cyclic peptide isolated from the marine-derived fungus *Aspergillus terreus* SCSGAF0162 which revealed the presence of d-Ala in its structure [108]. Nevertheless, it was not possible to distinguish between d-Ile and d-*allo*-Ile. Compound **104** showed promising inhibitory effects to the influenza virus strains A/WSN/33, and A/Hong Kong/8/68 (IC_50_ values of 15 and 8.1 µM, respectively) as well as cytotoxicity against U937 and MOLT4 cell lines (IC_50_ values of 6.5 and 6.2 µM, respectively) [108].

There are some reports describing the application of different types of CSPs, including crown ethers and macrocyclic antibiotics, for a chiral HPLC as the only method for analysis of the absolute configuration of the amino acids of peptides. Thus, the determination of the stereochemistry of the amino acids in the cyclic peptides sclerotides A (**105**) and B (**106**), isolated from the marine-derived fungus *Aspergillus sclerotiorum* PT06-1 [109], and cordyheptapeptides C–E (**107**–**109**), isolated from the marine-derived fungus *Acremonium persicinum* SCSIO 115 [110], was achieved via chiral HPLC analysis of the hydrolysates using the crown ether-based CSP Crownpak CR (+). Sclerotides A (**105**) and B (**106**) were found to comprise l-Thr, l-Ala, d-Phe, and d-Ser [109]. Moreover, the presence of *N*-Me-d-Gly, and l-Val in cordyheptapeptides C (**107**) and D (**108**) and *N*-Me-l-Gly, *N*-Me-d-Tyr, and l-*allo*-Ile in cordyheptapeptide E (**109**) was confirmed, in addition to the present of other amino acids common to the three cyclic peptides [110]. Sclerotides A (**105**) and B (**106**) displayed antifungal activity against *Candida albicans*, with MIC values of 7.0 and 3.5 µM, respectively. Furthermore, sclerotide B (**106**) also exhibited cytotoxicity against HL-60 cell line as well as antibacterial activity against *Pseudomonas aeruginosa* [109] whereas cordyheptapeptides C (**107**) and E (**109**) exhibited cytotoxic activity against SF-268 (IC_50_ values of 3.7 and 3.2 µM, respectively), MCF-7 (IC_50_ values of 3.0 and 2.7 µM, respectively), and NCI-H460 (IC_50_ values of 11.6 and 4.5 µM, respectively) tumor cell lines [110]. Recently, the macrocyclic antibiotic-based CSP Chirobiotic T was employed in our group to determine the stereochemistry of amino acid residues of a new cyclic hexapeptide, similanamide (**110**), isolated from a marine sponge-associated fungus *Aspergillus similanensis* KUFA 0013 [111] which confirmed the presence of l-Ala, d-Leu, l-Val and d-pipecolic acid as its amino acids constituent. By using a similar approach, the absolute configuration of all the amino acids of two new cyclotetrapeptides, sartoryglabramides A (**111**) and B (**112**), isolated from the marine sponge-associated fungus *Neosartorya glabra* KUFA 0702, were assigned to be L-configuration in both cyclic peptides [112]. Further details are described in the case-study presented below.

### 3.2. Cyclic Depsipeptides

**Figure 9:**
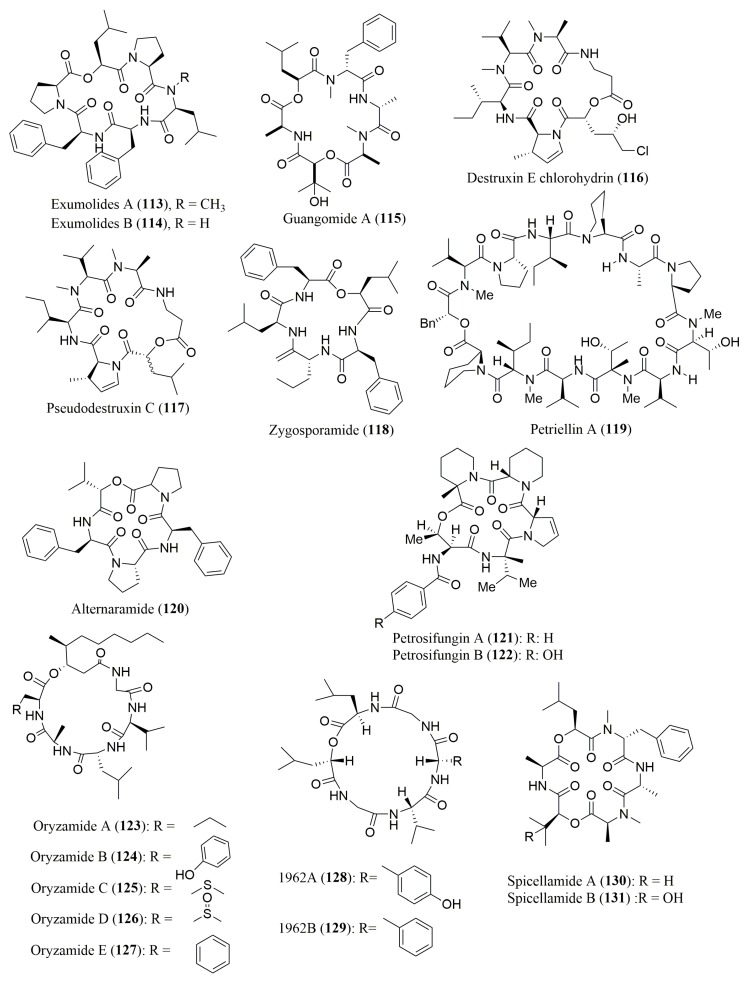
Structure of cyclic depsipeptides **113**–**131**, isolated from marine-derived fungi.

Most of the works describing the stereochemistry determination of amino acid residues of cyclic depsipeptides, isolated from marine fungus (Figure 9), employed Marfey’s method coupled with HPLC, using FDAA or FDLA as derivatization reagents (Table 5). 

The structures of exumolides A (**113**) and B (**114**), cyclic depsipeptides isolated from the marine fungus of the genus *Scytalidium*, were confirmed to have l-Pro, l-Phe and *N*-Me-l-Leu in their composition [113]. Moreover, guangomide A (**115**), isolated from an unidentified sponge-derived fungus, was found to comprise *N*-Me-d-Phe [114]. The absolute configuration of common amino acid residues in destruxin E chlorohydrin (**116**) and pseudodestruxin C (**117**), isolated from the marine-derived fungus *Beauveria felina*, indicated the presence of *N*-Me-l-Ala and l-Ile in **116**, l-Phe in **117**, and *N*-Me-l-Val in both cyclic depsipeptides [115]. Furthermore, the absolute configuration of amino acid residues in zygosporamide (**118**), isolated from the marine-derived fungus *Zygosporium masonii* [116], petriellin A (**119**), isolated from the coprophilous fungus *Petriella sordida* [117], alternaramdie (**120**), isolated from the marine derived fungus *Alternaria* sp. SF-5016 [118], petrosifungins A (**121**) and B (**122**), isolated from a *Penicillum brevicompac-tum* strain of the Mediterranean sponge *Petrosia ficiformis Poiret* [119], were also successfully determined by Marfey’s method coupled with HPLC. Zygosporamide (**118**) displayed cytotoxic activity against RXF 393 and SF-268 cancer cell lines, with mean values of GI-50 of 6.0 and <5.6 nM, respectively [116] whereas guangomide A (**115**) [114] and alternaramdie (**120**) [118] showed antibacterial activity against *Staphylococcus epidermidis* and *Staphylococcus aureus*, respectively.

In the last few years, ultra-high-pressure liquid chromatography (UHPLC) is becoming an essential technique for ultra-fast separations, since it offers many benefits, including high efficiency in short analysis time and low solvent consumption [120,121]. Thus, the absolute configuration of the amino acid residues of oryzamides A–E (**123**–**127**), isolated from the sponge-derived fungus *Nigrospora oryzae PF18*, was achieved by Marfey’s analysis with FDLA, combined with UHPLC [122].

Spicellamides A (**128**) and B (**129**), which were isolated from the marine-derived fungus *Spicellum roseum*, exhibited cytotoxicity against rat neuroblastoma B104 cell line, with an IC_50_ value of 6.2 µg/mL for spicellamide B (**129**) [123]. It is interesting to note that Marfey’s method was not suitable for the determination of the configuration of all amino acid residues of these two peptides. Therefore, a chiral HPLC approach was also employed, using a ligand exchange type CSP [123]. Furthermore, the chiral HPLC, using the crown ether-based CSP Crownpak CR (+), was used as the only method for determination of the configuration of the amino acids residues to confirm the presence of l-Tyr, l-Val, d-Leu, and (*S*)-*O*-Leu in the cyclic depsipeptides 1962 A (**130**) and B (**131**), isolated from the endophytic fungus *Kandelia candel* [124]. The cyclic depsipeptide 1962 A (**130**) exhibited growth inhibitory activity against the human breast cancer cell line, MCF-7, with IC_50_ of 100 µg/mL [124].

## 4. Peptides from Marine Sponges

Marine sponges are an important source of new metabolites from the marine environment [125]. They are considered one of the most prolific sources of novel bioactive compounds, such as terpenoids, alkaloids, macrolides, nucleoside derivatives, polyethers, fatty acids, sterols, peroxides and other numerous organic compounds [17,126]. In addition, cyclic peptides and depsipeptides have also been isolated from marine sponges. Most bioactive compounds from sponges displayed myriad of biological activities including anti-inflammatory, antibiotic, antitumor, antimalarial, antiviral, antifouling, and immuno- or neurosuppressive [127]. However, a significant number of marine natural products isolated from sponges were tested for the anticancer activity, and many of them were successfully undergoing to preclinical and clinical trials [126,128]. More recently, among bioactive compounds discovered from marine sponges, bioactive peptides have aroused attention of many researchers [8,17].

### 4.1. Cyclic Peptides

**Figure 10:**
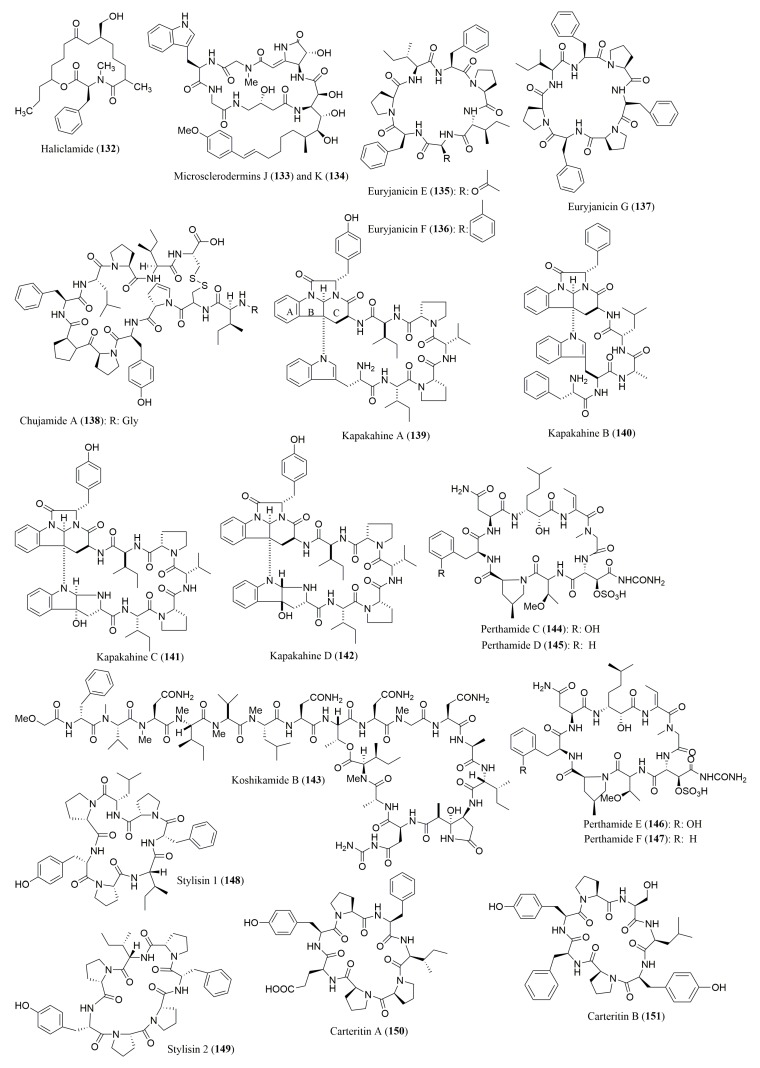
Structure of cyclic peptides **132**–**151**, isolated from marine sponges.

**Figure 11:**
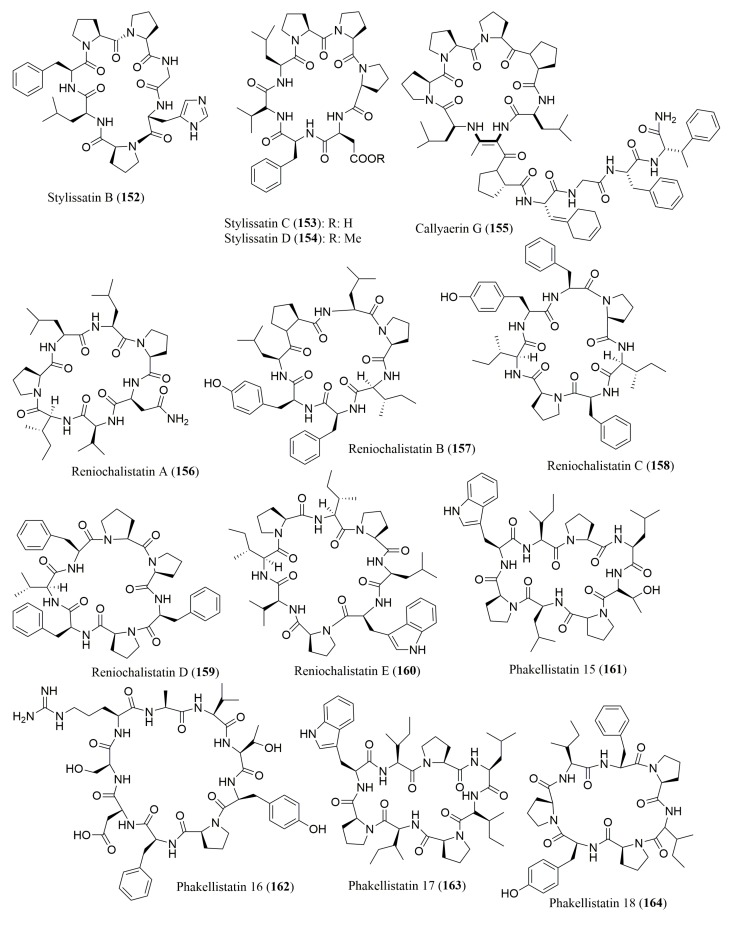
Structure of cyclic peptides **152**–**164**, isolated from marine sponges.

Several works reported the determination of the stereochemistry of the amino acid residues of diverse peptides isolated from marine sponges (Figure 10 and Figure 11), most of which described the application of Marfey’s method, using FDAA as the derivatization reagent (Table 6). By using Marfey’s method, Randazzo et al. [129] have showed that a 16-membered cyclic peptide, haliclamide (**132**), isolated from the Vanuatu marine sponge *Haliclona* sp., comprised the amino acid *N*-Me-l-Phe. The absolute configuration analysis of the amino acid residues of microsclerodermins J (**133**) and K (**134**), isolated from the sponge *Microscleroderma herdmani*, indicated, besides the amino acids common to both microsclerodermins, the presence of l-Phe, and l-Gly in **133**, and l-Val, and l-Ala in **134** [130]. Moreover, in the case of euryjanicins E–G (**135**–**137**), isolated from the Caribbean sponge *Prosuberites laughlini* [131], chujamide A (**138**), isolated from *Suberites waedoensis* [132], and kapakahines A–D (**139**–**142**), isolated from *Cribrochalina olemda* [133], all the amino acid residues were proved to have the L configuration. However, except for d-Phe, all the amino acid residues of koshikamide B (**143**), isolated from the marine sponge *Theonella* sp., were shown to possess L-configuration [134]. Furthermore, perthamides C–F (**144**–**147**), isolated from the sponge marine *Theonella swinhoei*, were found to comprise l-ThrOMe, and l-Phe; while perthamides C (**144**) and D (**145**) also comprise in their structures l-Asp, and (2*R*,3*S*)-βOHAsp [135,136]. Marfey’s method was also successfully used for evaluation of the stereochemistry of the amino acids of the cyclic peptides stylisins 1 (**148**) and 2 (**149**), stylissatins B–D (**152**–**154**), and carteritins A (**150**) and B (**151**), isolated from marine sponge S*tylissa* sp. [137,138,139], as well as of callyaerin G (**155**), isolated from the marine sponge *Callyspongia aerizusa* [140].

The marine sponge cyclic peptides whose configuration of their amino acids constituent was determined by Marfey’s method, were found to display interesting biological activities. For examples, haliclamide (**132**) exhibited cytotoxicity against NSCLC-N6 cell line, with an IC_50_ value of 4.0 µg/mL [129], while koshikamide B (**143**) showed growth inhibitory activity against P388 and HCT-116 cell lines, with IC_50_ values of 0.45 and 7.5 µg/mL, respectively [134]. Callyaerin G (**155**) also exhibited cytotoxicity against mouse lymphoma cell line (L5178Y), and HeLa cell line, with ED_50_ values of 0.53 and 5.4 µg/mL, respectively [140]. Moreover, perthamides C (**144**), D (**145**) and F (**147**) showed anti-inflammatory activity, with perthamide F (**147**) having a promising antipsoriatic effect [135,136].

The simultaneous application of Marfey’s method, using FDAA as derivatization reagent, and chiral HPLC, using a ligand exchange type CSP, afforded the total assignment of the configuration of all the amino acid residues of reniochalistatins A–E (**156**–**160**) [141]. Reniochalistatins A–E (**156**–**160**), the cyclic peptides isolated from the marine sponge *Reniochalina stalagmitis*, were found to have all the amino acid residues with l configuration, including l-Asn and l-Trp in reniochalistatins A (**156**) and E (**160**) respectively [141]. The octapeptide reniochalistatin E (**160**) exhibited cytotoxicity towards myeloma RPMI-8226, and gastric MGC-803 cell lines (IC_50_ values of 4.9 and 9.7 µM, respectively) [141].

Phakellistatins 15–18 (**161**–**164**) were analysed only by chiral HPLC, using the ligand exchange type Chirex 3126 d-penicillamine CSP, being able to identify that all the amino acids presented l-configuration. Furthermore, phakellistatins 15 (**161**) and 16 (**162**) exhibited cytotoxicity against P388 cancer cell line, with IC_50_ values of 8.5 and 5.4 µM, respectively, while phakellistatin 16 (**162**) was also active against BEL-7402 cancer cell line, with an IC_50_ value of 14.3 µM [142].

### 4.2. Cyclic Depsipeptides

**Figure 12:**
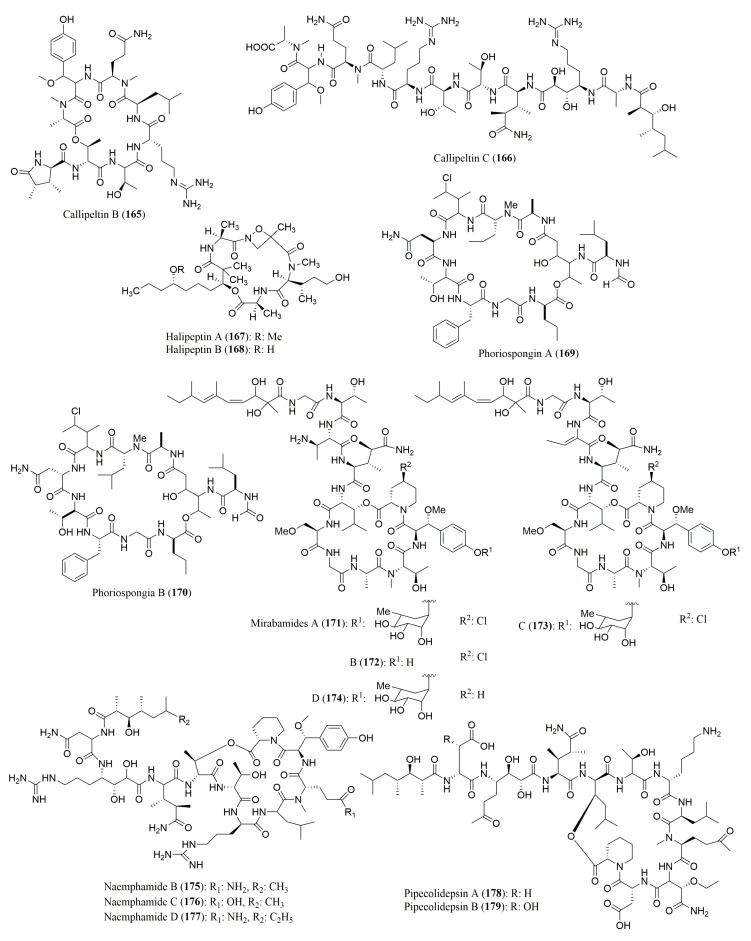
Structure of cyclic depsipeptides **165**–**179**, isolated from marine sponges.

**Figure 13:**
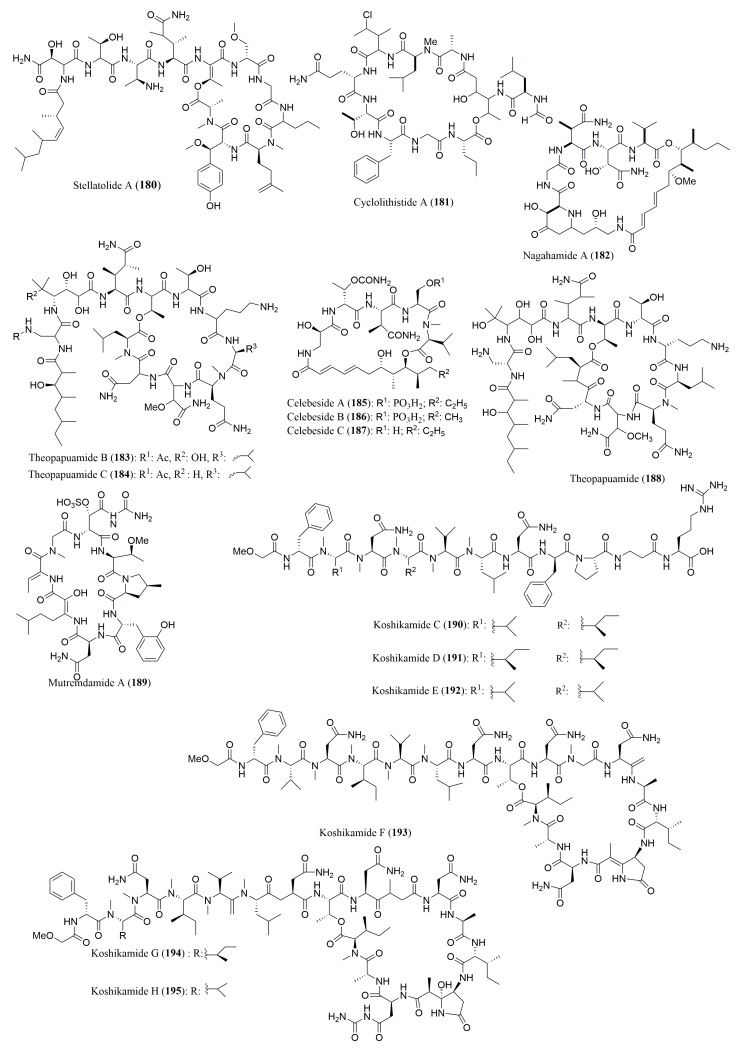
Structure of cyclic depsipeptides **180**–**195**, isolated from marine sponges.

A number of cyclic depsipeptides (Figure 12 and Figure 13), have been reported from marine sponges and Marfey’s method using FDAA as the derivatization reagent was the most used for the determination of absolute configuration of the amino acid residues. Table 7 gives some examples of the cyclic depsipeptides, isolated from marine sponges, whose stereochemistry of their amino acid residues was determined by Marfey’s method. By application of this method, callipeltins B (**165**) and C (**166**), isolated from the marine lithistida sponge *Callipelta* sp., were found to have in their structure l-Ala, *N*-Me-l-Ala, l-Leu, l-Thr and d-Arg [143]. For halipeptins A (**167**) and B (**168**), isolated from the marine sponge *Haliclona* sp.*,* the referred method was only able to determine the configuration for l-Ala [144]. Marfey’s method was successfully used to determine the absolute configuration of the amino acid constituents of several marine sponge cyclic peptides including phoriospongin A (**169**) and B (**170**), isolated from the marine sponges *Phoriospongia* sp. and *Callyspongia bilamellata* [145], mirabamides A–D (**171**–**174**), isolated from the marine sponge *Siliquarias-pongia mirabilis* [146], and neamphamides B–D (**175**–**177**), isolated from the marine sponge *Naemphius huxleyi* [147]. Furthermore, the stereochemistry determination of amino acid residues in pipecolidepsins A (**178**) and B (**179**), isolated from the marine sponge *Homophymia lamellose*, confirmed the presence of several l and d amino acid residues, besides the (3*S*,4*R*) diMe-l-Glu and (2*S*,3*S*)-EtO-Asp present in both peptides [148]. Stellatolide A (**180**), a cyclic depsipeptide isolated from *Ecionemia acervus*, was found to have *N*-Me-d-Ser and d-*allo*-Thr, among other l-configured amino acids [149]. The amino acid constituents of the cyclic depsipeptides cyclolithistide A (**181**) and nagahamide A (**182**), both isolated from the sponge *Theonella swinhoei*, were all found to have the *S* or l-configuration, and the 3-amino-5-hydroxybenzoic acid (AHBA) residue in nagahamide A (**182**) was established to have 3*S* configuration [150,151].

Almost all the cyclic peptides isolated from marine sponges displayed a variety of biological activities. Thus, callipeltin C (**166**) [143], cyclolithistide A (**181**) [150], and mirabamides A–D (**171**–**174**) [146] exhibited growth inhibitory activity against *Candida albicans*. Moreover, mirabamides A–D (**171**–**174**) also exhibited potent anti-HIV activities towards several HIV strains [146] whereas neamphamides B–D (**175**–**177**) displayed cytotoxic activity against several human cancer cell lines, including A549, HeLa, LNCaP, PC3, HEK, and NFF, with IC_50_ values ranging from 88 to 370 nM [147].

A simultaneous use of Marfey’s method and chiral HPLC analysis for stereochemical analysis of the amino acids of this type of peptides have been reported (Table 7). For examples, the absolute configuration of the amino acids of theopapuamides B (**183**) and C (**184**) and celebesides A–C (**185**–**187**), isolated from an Indonesian sponge *Siliquariaspongia mirabilis,* was successful assigned by HPLC-MS analysis of FDAA derivatives as well as via chiral HPLC analysis using a ligand exchange type CSP [152]. In the case of theopapuamide (**188**), isolated from a papua new Guinea Lithistid Sponge *Theonella swinhoei*, Marfey’s method was used to confirm the presence of d-*allo*-Thr, whereas chiral HPLC using a ligand exchange type CSP, revealed the presence of *N*-Me-l-Leu, d-Asp, l-Leu and *N*-Me-l-Glu in its structure [153]. The absolute configuration of the amino acid residues of a new sulfated cyclic depsipeptide, mutremdamide A (**189**) and six new highly *N*-methylated peptides, koshikamides C–H (**190**–**195**), isolated from different deep-water specimens of *Theonella swinhoei* and *Theonella cupola*, was also established by using both approaches. However, two different columns (C_12_ and C_18_) were used in Marfey’s method. By using chiral HPLC, it was possible to identify the amino acid residue *N*-Me-allo-l-Ile in koshikamide H (**195**) [154]. These cyclic peptides showed interesting biological activities. While theopapuamide (**188**) was cytotoxic against CEM-TART and HCT cell lines (IC_50_ values of 0.5 and 0.9 µM, respectively) [153], koshikamides F (**193**) and H (**195**) were active against a CCR5-using viral envelope, with IC_50_ values of 2.3 and 5.5 µM [154].

### 4.3. Lipopeptides

**Figure 14:**
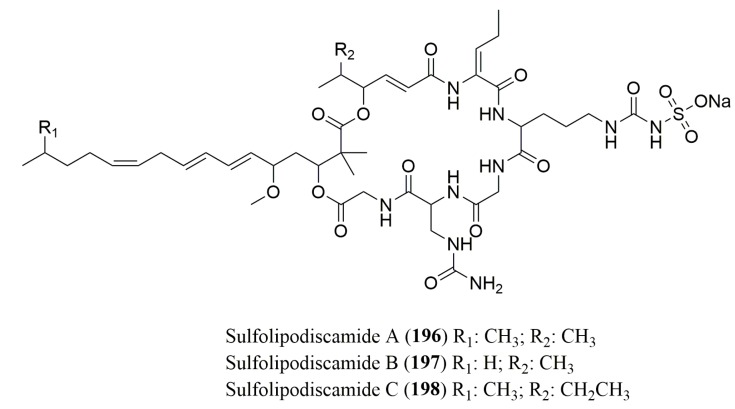
Structure of cyclic lipopeptides **196**–**198**, isolated from marine sponges.

The absolute configuration of the amino acids of new *N*-sulfoureidylated lipopeptides sulfolipodiscamides A–C (**196**–**198**), isolated from the *n*-butanol fraction of the marine sponge *Discodermia kiiensis* (Figure 14), was determined by Marfey’s method to be l-Uda and l-Gly (Table 8). Compound **196** was found to be cytotoxic against the murine leukemia cell line P388 with a IC_50_ value of 15 µM [155].

## 5. Peptides from Other Marine Invertebrates and Algae

A number of diverse bioactive peptides such as cyclic peptides, cyclic depsipeptides and linear peptides have been isolated from other marine invertebrates including ascidians, commonly called tunicates, mollusks, among others [17]. Moreover, the potential applications of many bioactive compounds from marine algae, mainly red and brown as well as some green algae, were reported [156].

### 5.1. Cyclic Peptides

**Figure 15:**
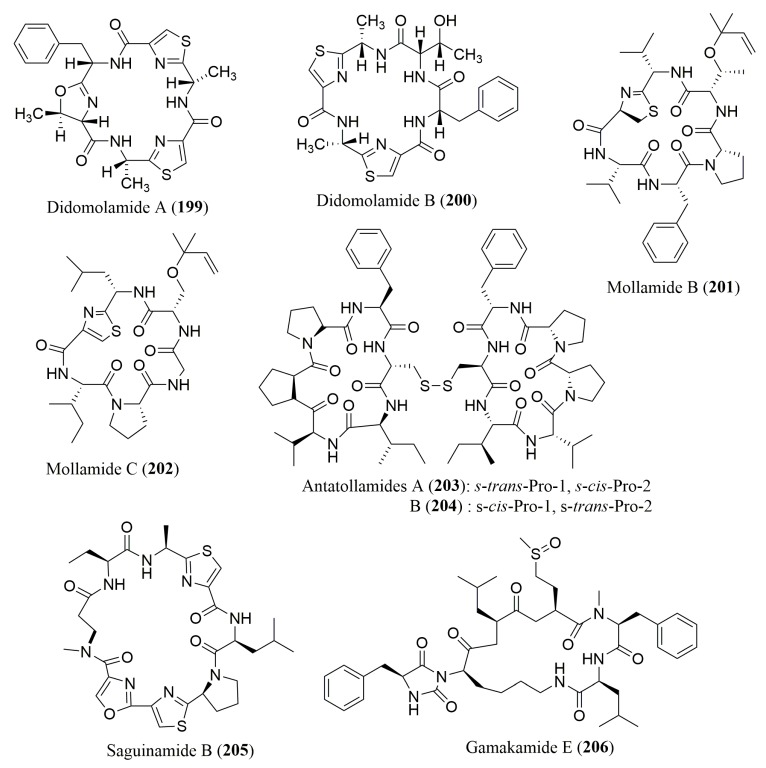
Structure of cyclic peptides **199**–**206**, isolated from marine invertebrates and marine algae.

To the best of our knowledge, only five works described the analysis of the stereochemistry of the cyclic peptides from marine invertebrates and algae (Figure 15). In all reported works, Marfey’s method was employed (Table 9). Among these, the determination of the absolute configuration of the cyclic hexapeptides didmolamides A (**199**) and B (**200**) and mollamides B (**201**) and C (**202**), isolated from the marine ascidian *Didemnum molle* from Madagascar and Indonesia, respectively, was performed by Marfey’s method using FDAA as derivatization reagent [157,158]. These compounds showed interesting biological activities, particularly, cytotoxicity against A549, HT29 MEL28 tumor cell lines, with IC_50_ values ranging from 10 to 20 µg/mL for didmolamides A (**199**) and B (**200**) [157] while **201** showed antimalarial activity against *Plasmodium falciprum*, clones D6 and W2, with IC_50_ values of 2.0 and 21 µg/mL, respectively [158].

Furthermore, the stereochemical determination of antatollamides A (**203**) and B (**204**), isolated from the marine ascidian *Didemnum molle*, sanguinamide A (**205**), isolated from the sea slug *Hexabranchus sanguineus*, and gamakamide E (**206**), isolated from the oysters *Crassostrea giga*, was carried out by Marfey’s method using FDLA as a derivatization reagent. The analysis demonstrated that most of their amino acids have the L-configuration, with the exception of d-Ala and d-Lys in antatollamides A (**203**) and B (**204**), and gamakamide E (**206**), respectively [159,160,161].

### 5.2. Cyclic Depsipeptides

**Figure 16:**
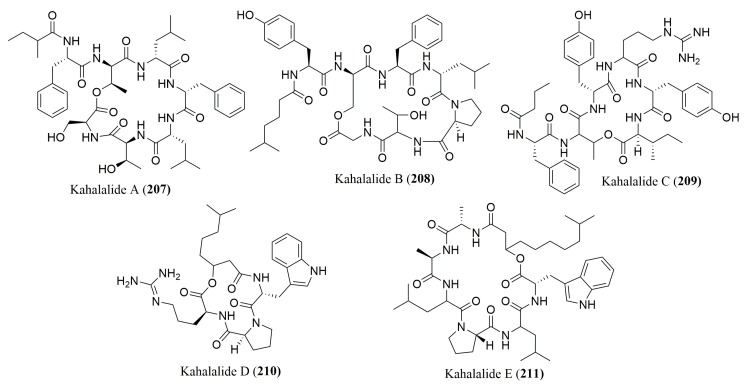

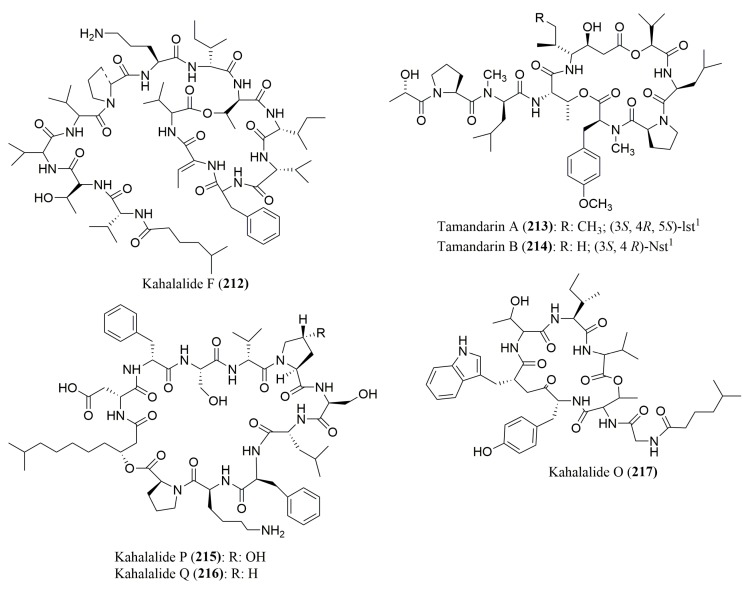
Structure of cyclic depsipeptides **207**–**217**, isolated from marine invertebrates and marine algae.

To the best of our knowledge, only four works reported the determination of the stereochemistry of amino acid constituents of the cyclic depsipeptides from marine invertebrates and algae (Figure 16). Among these, three employed only Marfey’s method, specifically for peptides **207**–**216**. However, for peptide **217**, Marfey’s method was not efficient and, as a consequence, a ligand exchange type CSP was also used for complete determination of the configuration of its amino acids (Table 10).

The determination of the absolute configuration of the amino acids in kahalalides A–F (**207**–**212**), isolated from the marine mollusk *Elysia rufescens*, was performed by using FDLA as the derivatization reagent and the presence of diverse residues of l- and d-Val in these peptides was confirmed [162]. Using FDAA as the Marfey derivatization reagent, the absolute configuration of tamandarins A (**213**) and B (**214**), isolated from an unidentified Brazilian marine ascidian of the family Didemnidae [163], and kahalalides P (**215**) and Q (**216**), isolated from green algae *Bryopsis* species [164] were elucidated. In the case of kahalalide O (**217**), the absolute configuration of its amino acid constituents was determined by Marfey’s method and chiral HPLC analysis, using a ligand exchange type CSP [165]. Tamandarin A (**213**) was found to display cytotoxicity against BX-PC3, DU-145, and UMSCC10b human cancer cell lines, with IC_50_ values of 1.79, 1.36, and 0.99 µg/mL, respectively [163].

### 5.3. Lipopeptides

**Figure 17:**
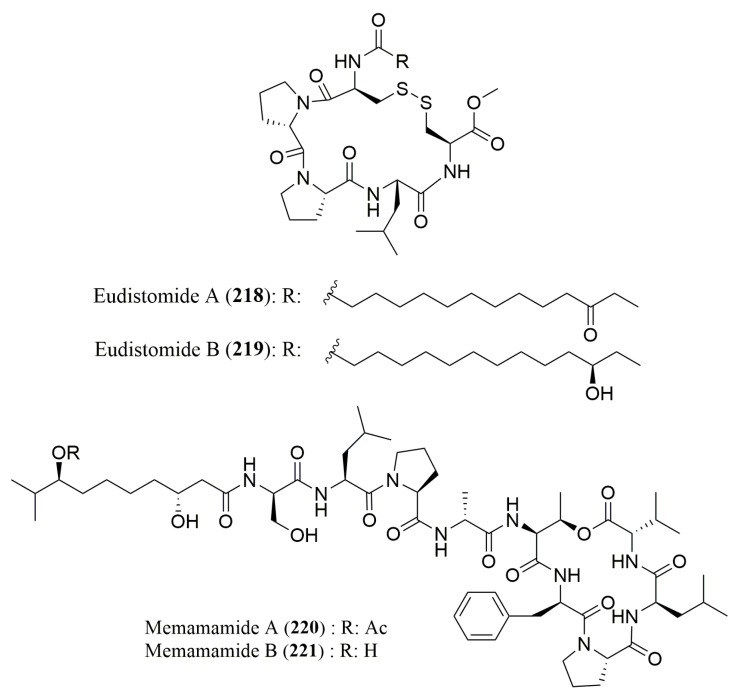
Structure of lipopeptides **218**–**221**, isolated from marine invertebrates and marine algae.

For lipopeptides isolated from other marine invertebrates and algae, there are only two works which reported the use of a chiral HPLC for the stereochemistry determination of the amino acid residues (Table 11) of the peptides **218**–**221** (Figure 17).

Chiral HPLC analysis by using a ligand exchange type CSP (Phenomenex Chirex Phase 3126) was used to determine the configuration of the amino acid residues in eudistomides A (**218**) and B (**219**), isolated from an ascidian *Eudistoma* sp. It was possible to verify the presence of l-Pro, l-Ala and l-Leu in both compounds as well as the presence of l-Cyp in eudistomide A (**218**) [166]. Similarly, a chiral HPLC analysis using a ligand exchange type CSP (CHIRALPAK (MA+)) was able to confirm the presence of four l-amino acid residues and d-Ala, d-Phe, and d-Ser in mebamamides A (**220**) and B (**221**), isolated from the green alga *Derbesia marina* [167].

## 6. Case-Study: Chiral HPLC in the Analysis of the Stereochemistry of Cyclopeptides Isolated from Marine Sponge-Associated Fungi

**Figure 18:**
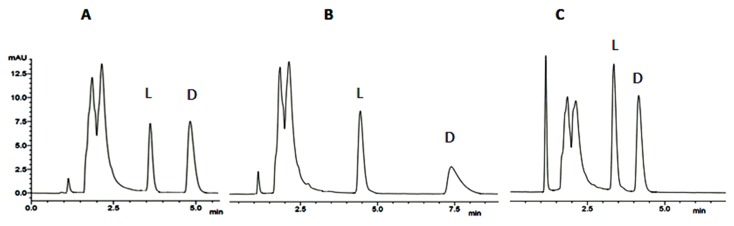
Chromatograms of enantiomeric mixture of dl-Ala (**A**), dl-pipecolic acid (**B**), and dl-Val (**C**). Column, Chirobiotic T; Mobile phase, MeOH:H_2_O:acetic acid (70:30:0.02 *v*/*v*/*v*); Flow rate, 1.0 mL/min; UV detection, 210 nm.

Recently, the determination of the stereochemistry of the amino acid residues of three bioactive marine natural products, by chiral HPLC analysis of their acidic hydrolysates, using appropriate d- and l-amino acid standards was achieved in our group [111,112]. The marine sponge-associated fungus *Aspergillus similanensis* KUFA 0013 was the source of the cyclohexapeptide similanamide (**110**) (Figure 8), while cyclotetrapeptides sartoryglabramides A (**111**) and B (**112**) (Figure 8) were isolated from the marine sponge-associated fungus *Neosartorya glabra* KUFA 0702. The enantioseparations of the amino acids were successfully performed on Chirobiotic T column under reverse phase elution conditions. Actually, the teicoplanin selector of this column has several characteristic features that make it suitable for amino acid analysis [168,169]. Figure 18 shows selected chromatograms of the enantioseparation of standard amino acids.

**Figure 19:**
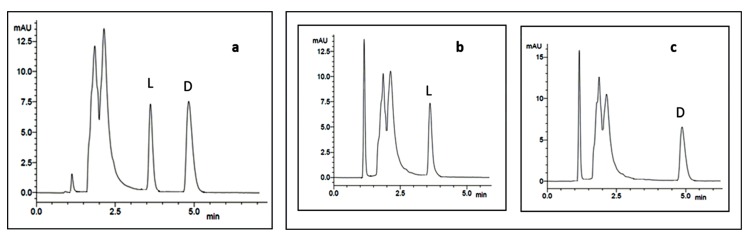
Chromatograms of enantiomeric mixture of dl-Ala (**a**), l-Ala (**b**), and d-Ala (**c**). Column, Chirobiotic T; Mobile phase, MeOH:H_2_O:acetic acid (70:30:0.02 *v*/*v*/*v*); Flow rate, 1.0 mL/min; UV detection, 210 nm.

The elution order of all the standard enantiomers of amino acids was confirmed by injecting solutions of the racemic or enantiomeric mixtures of amino acids and then each enantiomer separately. As an example, Figure 19 shows the chromatograms obtained during the method development for the determination of the elution order of Ala. As expected, the d-enantiomer was always more strongly retained than the corresponding l-enantiomer on Chirobiotic T column [168]. Mixed HPLC analyses of the acidic hydrolysates with appropriate standard amino acids (co-injection) (Table 12), confirmed the stereochemistry of the amino acids of the three cyclopeptides [111,112]. Chiral HPLC technique demonstrated to be decisive leading to the unambiguous elucidation of the amino acid constituents of the three marine natural products.

Additionally, the in vitro growth inhibitory activity against MCF-7, breast adenocarcinoma, NCI-H460, non-small cell lung cancer and A373, melanoma, cell lines, as well as antibacterial activity against reference strains and the environmental multidrug-resistant isolates (MRS and VRE) were evaluated for cyclopeptide **110**. Only weak activity against the three cancer cell lines was observed [111]. Moreover, cyclopeptides **111** and **112** were tested for their antifungal activity against filamentous (*Aspergillus fumigatus* ATCC 46645), dermatophyte (*Trichophyton rubrum* ATCC FF5) and yeast (*Candida albicans* ATCC 10231), as well as for their antibacterial activity against Gram-positive (*Escherichia coli* ATCC 25922) and Gram-negative (*Staphyllococus aureus* ATCC 25923) bacteria. None of them exhibited antibacterial or antifungal activities [112].

## 7. Conclusions

**Figure 20:**
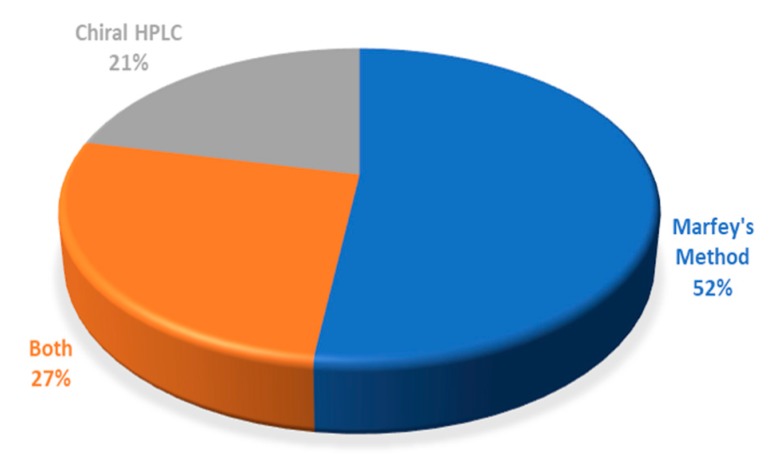
Distribution of the reported studies concerning the determination of the stereochemistry of marine peptides according to the methods used.

In summary, concerning all the reported studies surveyed in this review, which are related to the determination of the absolute configuration of the marine peptides, their distribution according to the methods used, is shown in Figure 20. It is possible to conclude that Marfey’s method is the most employed accounting for 52% of the reported studies, while only 21% of the studies described the use of chiral HPLC analysis. Moreover, 27% of the studies included the application of both methods. In fact, in some cases, the complementarity of both methods demonstrated to be crucial for the stereochemical analysis of all the amino acid residues.

**Figure 21:**
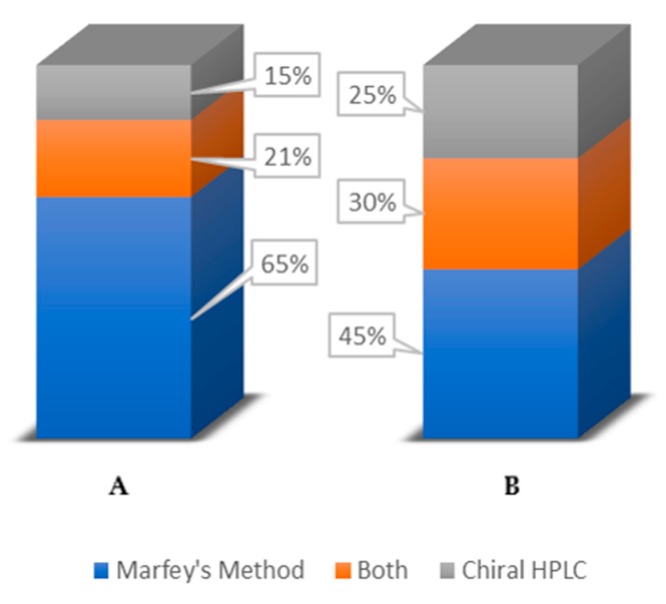
Distribution of the studies concerning the determination of the stereochemistry of marine peptides according to the method used before (**A**) and after 2007 (**B**).

Figure 21 compares the reported studies before and after 2007. Interestingly, it is possible to observe that in the last ten years, Marfey’s method is still the most used for determination of the absolute configuration of amino acid residues in marine peptides. However, it is important to point out a notable increase of the number of studies related to a chiral HPLC analysis, either as the only method or in a combination with Marfey’s method.

In our opinion, the current trend is to use chiral HPLC for stereochemical analysis due to many advantages of this method. For examples, there is no need for prior derivatization, it requires much less sample manipulation and the results are more rapid to obtain. In contrast, Marfey’s method involves time-consuming and labor-intensive procedure.

We believe that the reasons that can justify the actual low number of studies using chiral HPLC is due to the price of the commercially available CSPs and the fact that there is no universal CSP, i.e., one CSP can only separate a limited number of chiral compounds and, in many cases, the choice of CSP may become a very difficult task.

## Figures and Tables

**Table 1:** Cyclic peptides from marine cyanobacteria and other bacteria.

Peptide	Source	aa Composition	Chromatographic Conditions	Biological Activities	Refs.
Tetrapeptide (**1**)	Bacterium *Nocardiopsis* sp.	l-Ile, l-Leu, l-Pro	Marfey’s method (FDAA) combined with HPLC C_18_ (YMC-ODS-A) (4.5 × 250 mm) Flow rate: 0.8 mL/min; UV detection at 340 nm MP: ACN(aq) (0–50% (*v*/*v*)) with 0.1% TFA	Cytotoxicity toward the leukemia cell-line K-562	[60]
Anabaenopeptins NZ825, NZ841, NZ857 (**2**–**4**)	Cyanobacterium *Anabaena* sp.	l-Ile, d-Lys, l-Phe; **2**: l-Hph; **3**: l-Hph, l-Hty; **4**: l-Hty	Marfey’s method (FDAA) combined with HPLC Merck Chromolith performance RP-18e, (4.6 × 100 mm) MP: 50 mM TEAP buffer (pH 3)/ACN (9:1 to 1:1 *v*/*v*)	No inhibition of serine proteases	[61]
Aurilides B (**5**) and C (**6**)	Cyanobacterium *Lyngbya majuscula*	l-Val, *N*-Me-l-Ile, l-Ile	Ligand Exchange Type CSP; Phenomenex Chirex 3126 (D) (4.6 × 250 mm); Flow rate: 1.0 mL/min; UV detection at 254 nm; MP: 2 mM CuSO_4_ in ACN/H_2_O (5/95 *v*/*v*) or 2 mM CuSO_4_ in ACN/H_2_O (15/85 *v*/*v*)	Cytotoxicity against NCl-H460 and neuro-2a mouse neuroblastoma cell lines **5**: also active against leukemia, renal, and prostate cancer cell lines	[62]
		*N-*Me-l-Ala **6**: *N*-Me-l-*allo*-Ile, d-Hiva	Marfey’s method (FDAA) combined with HPLC Microsob-MV C_18_ (4.6 × 250 mm) Flow rate: 1.0 mL/min; UV detection at 254 nm MP: 50 mM TEAP buffer pH 3/ACN (9:1 to 1:1 *v*/*v*)		
Urukthapelstatin A (**7**)	Marine Derived *Mechercharimyces asporophorigenens* YM11-542	l-Ala	Marfey’s method (FDAA) combined with HPLC ODS-80Ts column (4.6 × 150 mm) Flow rate:1.0 mL/min; UV detection at 340 nm MP: MeOH, 0.1% TFA containing ACN or H_2_O	Growth inhibition of human lung cancer A549 cells, cytotoxicity against a human cancer cell line panel	[63,70]
		d-*allo*-Ile	Ligand Exchange Type CSPSumichiral OA-5000 column (4.6 × 150 mm) Flow rate: 1.0 mL/min; UV detection at 254 nm MP: 5% IPA containing 2 mM CuSO_4_		
Pompanopeptins A (**8**) and B (**9**)	Cyanobacterium *Lyngbya confervoides*	**8**: l-Val, l-Thr, l-Met (O), *S*-Ahp, l-Ile, l-Arg **9**: l-Ile	Ligand Exchange Type CSP; Phenomenex Chirex 3126 *N*,*S*-dioctyl-(d)-penicillamine, 5 µm (4.6 × 250 mm) Flow rate: 1.0 mL/min; UV detection at 254 nm MP: 2 mM CuSO_4_ or 2 mM CuSO_4_/ACN (95:5 *v*/*v*)	**8**: Trypsin inhibitory activity	[64]
		**8**: *N*,*O*-diMe-Br-l-Tyr	Marfey’s method (FDLA) combined with HPLC-MS Phenomenex Synergi 4u Hydro RP 80A (2 × 340 nm) Flow rate: 0.15 mL/min; UV detection at 254 nm MP: ACN/HCOOH (10–90:0.1 *v*/*v*) in gradient		
		**9**: d-Lys, l-Val, d-Glu	Marfey’s method (FDLA) combined with HPLC-MS Alltech Altima HP C_18_ HL 54 (250 × 4.6 mm) Flow rate: 1.0 mL/min; PDA detection from 200–500 nm MP: ACN/aq TFA (30–70:0.1 *v*/*v*) in gradient		
Marthiapeptide A (**10**)	Deep sea-derived *Marinactinospora thermotolerans* SCSIO 00652	l-Ile	Ligand Exchange Type CSP; MCIGELCR10W (4.6 × 150 mm); Flow rate: 0.5 mL/min; UV detection at 254 nm; MP: 2 mM CuSO_4_ solution	Antibacterial and cytotoxic activities	[65]
		d-Phe, l-Ile	Marfey’s method (FDAA) combined with HPLC Zorbax SB-C_8_ column, 5 μm (2.1 × 30 mm)		
Nocardiamides A (**11**) and B (**12**)	Marine-derived Actinomycete *Nocardiopsis* sp. CNX037	l-Tyr, d-Leu, d- and l-Val	Marfey’s method (FDAA or FDLA) combined with HPLC; Conditions not described	Antimicrobial activity and no cytotoxicity against HCT-116 cell line	[66]
		**11**: l-Ile	Ligand Exchange Type CSP; MCIGELCRS10W, (4.6 × 250 mm); Flow rate: 0.5 mL/min; UV detection at 254 nm; MP: 2 mM CuSO_4_/H_2_O		
Destomides B–D (**13**–**15**)	Deep sea-derived *Streptomyces scopuliridis* SCSIO ZJ46	l-Asn, d-Leu **13**: l-Trp, l-Val, l-Leu; **14**: l-Gly, l-Ile, **15**: l-Gly, l-Ile, l-Leu	Marfey’s method (FDAA) combined with HPLC Phenomenex ODS column, 5 µm (4.6 × 150 mm) Flow rate: 1.0 mL/min; UV detection at 340 nm MP: ACN:H_2_O:TFA (15:85:0.1 to 90:10:0.1)	**13**: Antimicrobial activity against *staphylococcus aureus* ATCC 29213, *Streptococcus pneumoniae* NCTC 7466 and MRSE shhs-E1 **13**–**15**: no cytotoxicity	[67]
		**15**: l-Kyn	Ligand Exchange Type CSP; MCIGELCRS10W column, 3 µm (4.6 × 50 mm); Flow rate: 0.5 mL/min; UV detection at 254 nm; MP: 2 mM CuSO_4_ aqueous solution		
Janadolide (**16**)	Cyanobacterium *Okeania* sp.	*N*-Me-l-Leu, l-Pro, l-Val	Ligand Exchange Type CSP; Diacel CHIRALPAK (MA+) (4.6 × 50 mm); Flow rate: 1.0 mL/min; UV detection at 254 nm; MP: 2.0 mM CuSO_4_	Antitrypanosomal activity	[68]
		*N*-Me-l-Ala	Marfey’s method (FDAA) combined with HPLC Cosmosil Cholester (4.6 × 50 mm); Flow rate: 1.0 mL/min; UV detection at 340 nm MP: 0.02 M NaOAc(aq)/MeOH (45/55 *v*/*v*)		
Wewakazole B (**17**)	Cyanobacterium *Moorea producens*	l-Ala, l-Phe, l-Pro	Macrocyclic Antibiotic type CSP Chirobiotic TAG (2.1 × 250 mm); Flow rate: 0.3 mL/min; UV detection at 340 nm; MP: 0.1% aq. HCOOH and 1% (*v*/*v*) NH_4_OAc in MeOH	Cytotoxicity against MCF7 and human 460 lung cancer cell lines	[69]
		l-Ile	Ligand Exchange type CSP; Sumichiral OA-5000 (4.6 × 150 mm); Flow rate: 1.0 mL/min; UV Detection at 254 nm; MP: MeOH/2.0 mM CuSO_4_ in H_2_O (5/95 *v*/*v*)		

aa—Amino acid; FDAA—1-Fluoro-2-4-dinitrophenyl-5-l-alanine amide; ESI—Electrospray Ionization; LC—Liquid Chromatography; MS—Mass spectrometry; HPLC—High Performance Liquid Chromatography; MP—Mobile Phase; TEAP—Triethylammonium phosphate; ACN—Acetonitrile; CPA—Carboxypeptidase A; TFA—Trifluoracetic acid; MeOH—Methanol; TEA—Triethylamine; IPA—Isopropyl alcohol; FDLA—1-fluoro-2-4-dinitrophenyl-5-d,l-leucine amide; NaOAc—Sodium acetate; NH_4_OAc—Ammonium acetate.

**Table 2:** Cyclic depsipeptides from marine cyanobacteria and other bacteria.

Peptide	Source	aa Composition	Chromatographic Conditions	Biological Activities	Refs.
Malevamides B (**18**) and C (**19**)	Cyanobacterium *Symploca laete-viridis*	l-Pro, *N*-Me-l-Val, *N*-Me-l-Phe **18**: l-Ile, *N*-Me-l-Ala, *N*-Me-d-Val, l-Val, (*R*)-Hiva; **19**: l-Ala, *N*-diMe-l-Ser, l-Leu, *N*-Me-d-Ala, *N*-Me-l-Ile, (*S*)-Hiva	Ligand Exchange Type CSP; Chirex (D) Penicillamine, Phenomenex 00G-3126E0 (4.6 × 250 mm) MP: 1.7 mM CuSO_4_ in ACN/H_2_O (14:86 *v*/*v*), 1.9 mM CuSO_4_ in ACN/H_2_O (5:95 *v*/*v*) or 2.0 mM CuSO_4_ in H_2_O Flow rate: 1.0 and 0.8 mL/min; UV detection at 245 nm	Inactive against P-388, A-549 and HT-29 cancer cells	[71]
Lyngbyapeptin B (**20**)	Cyanobacterium *Lyngbya majuscula*	*N*-Me-l-Ile, *N*-Me-l-Leu, *N*,*O*-diMe-l-Tyr	Ligand Exchange Type CSP; Chirex (D) Penicillamine, Phenomenex 00G-3126E0 (4.6 × 250 mm) MP: 2 mM CuSO_4_ Flow: 0.8 mL/min; UV detection at 254 nm	Cytotoxicity against KB and LoVo cells	[72]
Tasipeptins A (**21**) and B (**22**)	Cyanobacterium *Symploca* sp.	l-Thr, l-Val, l-Leu, l-Glu, *N*-Me-l-Phe	Ligand Exchange Type CSP; Phenomenex Chirex Phase 3126 (D) (4.6 × 250 mm) MP: 2 mM CuSO_4_; 2 mM CuSO_4_/ACN (95:5 or 85:15 *v*/*v)* UV detection at 254 nm	Cytotoxicity toward KB cells	[73]
Wewakamide A (**23**)	Cyanobacteria *Lyngbya semiplena* and *Lyngbya majuscula*	l-M-Ala, l-Pro, l-Val, l-Me-Leu, l-Phe, l-Me-ILe, l-Hiv	Ligand Exchange Type CSP; Phenomenex Chirex 3126 (D) (4.6 × 250 mm); MP: 2 mM CuSO_4_ in H_2_O or 2 mM CuSO_4_ in ACN/H_2_O (15:85 or 5:95 *v*/*v*) Flow rate: 0.7, 0.8, 1.0 mL/min; UV detection at 254 nm	Brine shrimp toxicity	[74]
Cocosamide A (**24**) and B (**25**)	Cyanobacterium *Lyngbya majuscula*	l-Pro, l-Val, *N*-Me-l-Phe	Ligand Exchange Type CSP; Phenomenex Chirex (D), Penicillamine, 5 µm (4.6 × 250 mm) MP: 2.0 mM CuSO_4_/ACN (85:15 or 90:10 *v*/*v*) Flow rate: 1.0 mL/min; UV detection at 254 nm	Cytotoxicity against MCF-7 (breast cancer) and HT-29 (colon cancer) cells	[75]
Dudawalamides A–D (**26**–**29**)	Cyanobacterium *Moorea producens*	l-Dhoya, l-Hiva, l-Val **29**: d-allo-Hiva	Ligand Exchange Type CSP; Chirex Phase 3126 (D) 5 µm (4.6 × 250 mm); MP: 2 mM CuSO_4_-ACN (95:5 or 85:15 *v*/*v* or 87.5:12.5 *v*/*v*/*v*), ACN-H_2_O-HCOOH (30:70:0.1 or 70:30:0.1 *v*/*v*/*v*) Flow rate: 0.8 mL/min; UV detection at 340 nm	Antiparasitic activity	[76]
Pitipeptolides A (**30**) and B (**31**)	Cyanobacterium *Lyngbya majuscula*	l-Gly, l-Pro, l-Val, l-Ile, *N*-Me-l-Phe, (2*S*,3*S*)-Hmp	Ligand Exchange Type CSP; Chiralpak MA (+) (4.6 × 50 mm); MP: 2 mM CuSO_4_: ACN (90:10 or 85:15 *v*/*v*) Flow rate: 1.0 mL/min; UV detection at 254 nm	Cytotoxic, antimycobacterial and elastase inhibitory activities	[77]
Kohamamides A–C (**32**–**34**)	Cyanobacterium *Okeania* sp.	l-Pro, l-Ala, l-Val, *N*-Me-l-Val, l-Leu; **32**: l-Ile	Ligand Exchange Type CSP; Chiralpak MA (+) (4.6 × 250 mm); MP: 2 mM CuSO_4_, ACN: 2 mM CuSO_4_ (15:85 *v*/*v*); Flow rate: 1.0 mL/min; UV detection at 254 nm	No growth inhibition against HeLa and HL60 cells	[78]
Marformycins A–F (**35**–**40**)	Deep sea-derived *Streptomyces drozdowiczii*	**35**: d-allo-Ile, l-Val; **36**: d-allo-Ile, l-allo-Ile; **37**: d-Val, l-allo-Ile; **38**: d-allo-Ile, l-allo-Ile, l-Leu; **39** and **40**: l-Thr, l-Val, d-Val, l-Leu	Ligand Exchange Type CSP; MCIGELCRS10W (4.6 × 50 mm); MP: 2 mM CuSO_4_ in H_2_O Flow rate: 0.5 mL/min; UV detection at 254 nm	Anti-infective activity against *Micrococcus luteus*	[79]
Pitiprolamide (**41**)	Cyanobacterium *Lyngbya majuscula*	l-Pro, l-Val	Macrocyclic Antibiotic Type CSP; Chirobiotic TAG (4.6 × 250 mm); MP: MeOH/10 mM NH_4_OAc (40:60 *v*/*v*) *(*pH 5.6) Flow rate: 0.5 mL/min	Cytotoxicity against CT116 and MCF7 cancer cell lines and antibacterial activity	[80]
Palau’amide (**42**)	Cyanobacterium *Lyngbya* sp.	l-Ala, l-Ile, *N*-Me-l-Ala, *N*-Me-d-Phe and d-hydroxyisocaproic acid	Ligand Exchange Type CSP; Phenomenex Chirex Phase 3126 (D) (4.6 × 250 mm) MP: 1 mM CuSO_4_; 2 mM CuSO_4_/ACN (95:5 or 85:15 *v*/*v)* Flow rate: 0.8 mL/min; UV detection at 254 nm	Cytotoxicity against KB cell line	[81]
Pitipeptolides C–F (**43**–**46**)	Cyanobacterium *Lyngbya majuscula*	l-Pro, l-Val, l-Ile, l-Phe, *N*-Me-l-Phe	Macrocyclic Antibiotic Type CSP; Chirobiotic TAG (4.6 × 250 mm); MP: MeOH/10 mM NH_4_OAc (40:60 *v*/*v*) *(*pH 5.6); Flow rate: 0.5 mL/min Detection by EIMS in positive ion mode (MRM scan)	**46**: Active against *Mycobacterium tuberculosis*	[82]
Ulongapeptin (**47**)	Cyanobacterium *Lyngbya* sp.	l-lactic acid, l-Val, *N*-Me-l-Val, *N*-Me-d-Val, *N*-Me-d-Phe	Ligand Exchange Type CSP; Phenomenex Chirex Phase 3126 (D), 4.6 × 250 mm MP: 2 mM CuSO_4_; 2 mM CuSO_4_/ACN (95:5 or 85:15 *v*/*v*) Flow rate: 1.00 mL/min; UV detection at 254 nm	Cytotoxicity against KB cells	[83]
		l-Val, *N*-Me-l-Val, *N*-Me-d-Val	Marfey’s method (FDLA) combined with HPLC YMC-Pack AQ-ODS (10 × 250 mm); MP: 50% ACN in 0.01 N TFA Flow rate: 2.5 mL/min; UV detection at 254 nm		
		2-hydroxy-3-methylvaleric acid *N*-Me-l-Ala	Ligand Exchange Type CSP; CHIRALPAK MA (+) (4.6 × 50 mm); MP: 1 mM CuSO_4_; 2 mM CuSO_4_/ACN (95:5 or 85:15 *v*/*v*) Flow rate: 0.7 mL/min; UV detection at 254 nm		
Largamides A–H (**48**–**55**)	Cyanobacterium *Oscillatoria* sp.	**48**: l-Val, l-Thr, l-Ala, l-Leu, d-Gln, d-Tyr; **49**: l-Val, l-Thr, l-Ala, l-Ahppa, d-Gln, d-Tyr; **50**: l-Val, l-Thr, l-Ala, l-Ahpha, d-Gln, d-Tyr; **51**: l-Val, l-Thr, l-Ala, l-Leu, l-Ahp, *N*-MeBr-l-Tyr, l-Ahppa; **52**: l-Val, l-Thr, l-Ala, l-Leu, l-Ahp, *N*-MeCl-l-Tyr; **53**: l-Val, l-Thr, l-Ala, l-Tyr, l-Ahp, *N*-MeCl-l-Tyr; **54**: l-Val, l-Thr, l-Ala, l-hTyr, l-Ahp, *N*-MeCl-l-Tyr; **55**: l-Val, l-Thr, l-Ala, l-Amppa, l-Gln, *N*-Me-l-Asn	Marfey’s method (FDLA) combined with HPLC Phenomenex Jupiter Proteo C_12_ column, 4 µm (4.6 × 150 mm); MP: ACN containing 0.01 M TFA Flow 0.5 mL/min; UV detection at 254 nm	**51**–**54**: Chymotrypsin inhibition	[84]
		d-Glyceric acid	Ligand Exchange Type CSP; Phenomenex Chirex 3126 (D) (4.6 × 150 mm); MP: 2 mM CuSO_4_:ACN (90/10 *v*/*v*); Flow 0.5 mL/min; UV detection at 254 nm		
Trungapeptins A–C (**56**–**58**)	Cyanobacterium *Lyngbya majuscula*	l-Val, l-*N*-MeVal, l-alloLeu, l-Pro	Marfey’s method (FDLA) combined with HPLC. Alltech Econosil C_18_; MP A:40% ACN with 0.04%TFA. MP B: 37.5% ACN with 0.05%TFA. Flow rate: 1.0 mL/min; UV detection at 254 nm	Brine shrimp toxicity and ichthyotoxicity	[85]
		Phenyllactic acid (*S*)	Ligand Exchange Type CSP; CHIRALPAK MA (+) (4.6 × 50 mm); MP: 2 mM CuSO_4_/ACN (85:15) Flow rate: 0.5 mL/min; UV detection at 254 nm		
Carriebowmide (**59**)	Cyanbacterium *Lyngbya polychroa*	l-Ala, *N*-Me-l-Leu, *N*-Me-d-Phe, l-Phe, l-Met	Ligand Exchange Type CSP; Phenomenex, Chirex (D) Penicillamine, 5μm (4.6 × 250 mm) MP: 2.0 mM CuSO_4_-ACN (95:5, 90:10, or 85:15 *v*/*v*) Flow rate: 0.8 or 1.0 mL/min; UV detection at 254 nm	Lipophilic extract reduced feeding on agar food pellets	[86]
		*R*-Hmba	Ligand Exchange Type CSP; Chiralpak MA (+) (4.6 × 250 mm); MP: 2.0 mM CuSO_4_-ACN (90:10 *v*/*v*) Flow rate:1.0 mL/min; UV detection at 254 nm		
		l-Aba	Ligand Exchange Type CSP; Phenomenex, Chirex (D) Penicillamine, 5 μm (4.6 × 250 mm); MP: 2.0 mM CuSO_4_ Flow rate:1.0 mL/min; UV detection at 254 nm		
		(2*R*,3*R*)-Amha	Marfey’s method (FDAA) combined with HPLC Atlantis, C_18_, (3.0 × 250 mm); MP: 50 mM NH_4_COOCH_3_(aq)-ACN (70:30 *v*/*v*) Flow rate: 1.0 mL/min; UV detection at 254 nm		
Symplocamide A (**60**)	Cyanobacterium *Symploca* sp.	l-Val, l-Thr, l-Ile, l-Cit, l-Gln, l-Btyr, l-But	Marfey’s method (FDAA) combined with HPLC Phenomenex Jupiter C_18_ column (4.6 × 250 mm) MP: ACN:H_2_O:HOAc (15:85:0.02 to 1:1:0.02 *v*/*v*/*v*) Flow rate: 0.5 mL/min; UV detection at 340 nm	Cytotoxicity and antimicrobial activities Chymotrypsin inhibitor	[87]
Kempopeptins A (**61**) and B (**62**)	Cyanobacterium *Lyngbya* sp.	**61**: *N-O*-diMe-Br-l-Tyr	Marfey’s method (FDLA) combined with HPLC Conditions not described	**61**: Elastase and chymotrypsin inhibition **62**: Trypsin inhibition	[88]
		**61**: *N*-Me-l-Tyr, l-Val, l-Thr-2, l-Pro, l-Phe, l-Ahp, l-Leu **62**: l-Lys, l-Thr, l-Val, l-Ile	Ligand Exchange Type CSP; Phenomenex Chirex Phase 3126 *N*,*S*-dioctyl-(d)-penicillamine column, 5 μm (4.6 × 250 mm); MP: 2 mM CuSO4 in H_2_O:ACN (95:5 *v*/*v*) or 2 mM CuSO_4_ Flow rate: 1.0 mL/min; UV detection at 254 nm		
Tiglicamides A–C (**63**–**65**)	Cyanobacterium *Lyngbya confervoides*	l-Ala, l-Thr, l-Val, d-Glu, d-Tyr; **63**: l-Htyr; **65**: l-Met (*O*)	Ligand Exchange Type CSP; Phenomenex, Chirex 3126, 5 μm (4.6 × 250 mm); Mobile Phase: 2 mM CuSO_4_ Flow rate: 1.0 mL/min; UV detection at 254 nm	Porcine pancreatic elastase inhibition	[89]
		**65**: l-Phe	Marfey’s method (FDLA) combined with HPLC Alltech Alltima HP C_18_, 5μm (4.6 × 250 mm) MP: 50–100% MeOH in 0.1% (*v*/*v*) aqueous TFA Flow rate: 0.8 mL/min; PDA detection at 200–500 nm		
Hantupeptin B (**66**)	Cyanobacterium *Lyngbya majuscula*	l-Pro, l-Val, *N*-Me-l-Val, *N*-Me-l-Ile	Marfey’s method (FDAA) combined with HPLC Phenomenex, Luna, 5 µm, (2.0 × 150 mm); MP: ACN in 0.1% (*v*/*v*) aqueous HCOOH; Flow rate: 0.2 mL/min	Cytotoxicity against MOLT-4 (leukemic) and MCF-7 (breast cancer) cell lines	[90]
		l-3-phenyllactic acid (*S*)	Ligand Exchange Type CSP; Chiralpak MA (+) (4.6 × 500 mm) MP: 2 mM CuSO_4_/ACN (85:15 *v*/*v*) Flow rate: 0.7 mL/min; UV detection at 218 nm		
Palmyramide A (**67**)	Cyanobacterium (*Lyngbya majuscula*) and a red alga *Centroceras* sp. complex	l-Val, *N*-Me-l-Val, l-Pro	Marfey’s method (FDAA) combined with HPLC/MS on a Merck LiChrospher 100 RP-18 (4.0 × 125 mm) MP: ACN:H_2_O:HCOOH (30:70:0.1 to 70:30:0.1 *v*/*v*/*v*) or 2.0 mM CuSO_4_ in H_2_O Flow rate: 0.7 mL/min; UV detection at 254 nm	Sodium channel blocking activity in neuro-2a cells and cytotoxic activity in H-460 (human lung carcinoma) cells	[91]
		l-Lac, l-Pla	Ligand Exchange Type CSP; Phenomenex Chirex 3126 (4.6 × 250 mm); Conditions not described		
Veraguamides A–G (**68**–**74**)	Cyanobacterium *Symploca* cf. *hydnoides*	**68**–**71**, **73** and **74**: l-Val, *N*-Me-l-Val, l-Pro; **70**: (2*S*,3*R*) Br-Hmoya; **71**: *N*-Me-l-Ile; **72**: l-Ile, *N*-Me-l-Val, *N*-Me-l-Ile, l-Pro	Macrocyclic Antibiotic Type CSP; Chirobiotic TAG (4.6 × 250 mm); MP: MeOH/10 mM NH_4_OAc (40:60 *v*/*v*) *(*pH 5.6); Flow rate: 0.5 mL/min	Cytotoxic activity against HT29 (colorectal adenocarcinoma) and HeLa (cervical carcinoma) cell lines	[92]
		**74**: 2*S*:3*R* dpv 2*R*:3*R* Dml	Marfey’s method (FDAA) combined with HPLC-MS Phenomenex Synergi Hydro-RP (4.6 × 150 mm) MP: MeOH:H_2_O:HCOOH (40–100% MeOH: 0.1% HCOOH); Flow rate: 0.5 mL/min		
Porpoisamides A (**75**) and B (**76**)	Cyanobacterium *Lyngbya* sp.	**75** and **76**: l-Ala, l-Pro, *N*-Me-d-Phe, (2*S*,3*S*)*-*Hmpa	Ligand Exchange Type CSP; Phenomenex Chirex 3126 (4.6 × 250 mm); MP: 5% or 15% ACN in 2 mM CuSO_4_ in H_2_O; Flow rate:1.0 mL/min	Cytotoxicity against HCT 116 (colorectal carcinoma) and U2OS (osteosarcoma) cells	[93]
		**75**: (2*S*,3*R*)-Amoa **76**: (2*R*,3*R*)-Amoa	Ligand Exchange Type CSP; Chiralpak MA (+) (4.6 × 50 mm); MP: 15% ACN in 2 mM CuSO_4_ in H_2_O Flow rate: 1.0 mL/min		
		(2*R*,3*R*) 3-NH_2_-2-Me-octanoic acid	Marfey’s method (FDAA) combined with HPLC YMC-Pack AQ-ODS (10 × 250 mm) MP: ACN:H_2_O: *N*-TFA (57:43:0.1 *v*/*v*/*v)* Flow rate: 2.5 mL/min; UV detection at 340 nm		
		**76**: (2*S*)-Hiva	Ligand Exchange Type CSP; CHIRALPAK MA (+) (4.6 × 50 mm); MP: ACN/2 mM CuSO_4_ (10:90 *v*/*v*) Flow: 1.0 mL/min; UV detection at 254 nm		
Companeramides A (**77**) and B (**78**)	Cyanobacterial assemblage collected from Coiba National Park, Panama	**77**: l-Ala, *N*-Me-l-Ala, l-Pro, l-Ile, *N*-Me-l-Leu, and *N*-Me-l-Val; **78**: l-Pro, *N*-Me-l-Val, l-Val, l-Ile, d- and *N*-Me-l-Ala	Marfey’s method (FDAA) combined with HPLC C_18_ column (3.9 × 150 mm) MP: 40 mM NH_4_OAc (pH 5.2):ACN (9:1 to 1:1 *v*/*v*) Flow rate: 1.0 mL/min; UV detection at 340 nm	Antiplasmodial activity against *Plasmodium falciparum*	[94]
		*S-*Hiva	Ligand Exchange Type CSP; Phenomenex Chirex 3126 (D) (4.6 × 250 mm); MP: CuSO_4_/ACN Flow: 1.0 mL/min; UV detection at 254 nm		
Piperazimycins A–C (**79**–**81**)	Fermentation broth of a *Streptomyces* sp.	(*S*)-AMNA, (*S*,*S*)-OHPip1, (*R*,*R*)-γOHPip2, **79**: (*S*)-αMeSer	Marfey’s method (FDAA) combined with HPLC C_18_; MP: ACN in H_2_O (10–100%) Flow rate: 1.0 mL/min; UV detection: 210, 254, 340 nm	**79**: Active against diverse cancer cell lines	[95]
Grassypeptolides D (**82**) and E (**83**)	Red sea cyanobacterium *Leptolyngbya* sp.	d-allo-Thr, *N*-Me-d-Leu, l-Thr, *N*-Me-l-Leu	Marfey’s method (FDAA) combined with HPLC Gemini C_18_ 110 A, 5 µm (4.6 × 250 mm)	Cytotoxicity against HeLa and mouse neuro-2a blastoma cells	[96]
		l-PLa, *N*-Me-l-Val, l-Pro, *N*-Me-l-Phe, (2*S*)-MeCysA, d-Aba, l-Cya, (2*R*,3*R*)-Maba	Marfey’s method (FDAA) combined with HPLC Kinetex XB-C_18_, 110 A, 2.6 µm (4.6 × 100 mm) MP: ACN:H_2_O:HCOOH (30:70:0.1 to 70:30:0.1 *v*/*v*/*v*) or ACN:H_2_O:TFA (30:70:0.1 to 70:30:0.1 *v*/*v*/*v*); Flow rate: 0.2 mL/min; UV detection at 340 nm and ESIMS		
Fijimycins A–C (**84**–**86**)	Fermentation broth of *Streptomyces* sp. strain CNS-575	**84**: d-PhSar, l-Ala, l-DiMe-Leu, Sar, d-Hyp, d-Leu, l-Thr; **85**: l-*N-*MeLeu, l-Ala, l-DiMeLeu, Sar, d-Hyp, d-Leu, l-Thr; **86**: l-PhSar, l-Ser, l-DiMeLeu, Sar, d-Hyp, d-Leu, l-Thr	Marfey’s method (FDAA) combined with HPLC C_18_ column, Luna (4.6 × 100 mm) MP: ACN:H_2_O:TFA (10:90:1 to 50:50:1 *v*/*v*/*v*) Flow rate: 0.7 mL/min; UV detection at 340 nm	Antibacterial activity against three MRSA strains of *Staphylococcus aureus*	[97]
Itralamides A (**87**) and B (**88**), and Carriebowmide sulfone (**89**)	Cyanobacterium *Lyngbya majuscula*	**87**: l-Ala, d-Ala, *N*-Me-l-Ala, *N*-Me-d-Phe, *N*-Me-l-Thr, *N*-Me-l-Val	Marfey’s method (FDLA) combined with HPLC Eclipse XDB-18, Agilent (4.6 × 150 mm) MP: ACN:H_2_O:HCOOH (20:80:0.1 to 80:20:0.1 *v*/*v*/*v*) Flow rate: 0.8 mL/min; Detection by ESI-MS	**88**: Cytotoxicity against HEK293 (human embryonic kidney) cell line	[98]
		**88**: *N*-Me-l-Ala, *N*-Me-d-Phe, *N*-Me-l-Thr, d-Val	Marfey’s method (FDLA) combined with HPLC Luna C18, Phenomenex, 5 µm (4.6 × 250 mm) MP: ACN:H_2_O:HCOOH (20:80:0.1 to 90:10:0.1 *v*/*v*/*v*) Flow rate: 0.8 mL/min		
		**89**: (2*S*,3*R*)-AMHA	Marfey’s method (FDLA) combined with HPLC-PDA dC_18_, 5 µm (3.0 × 250 mm); MP: ACN:H_2_O:HCOOH (0:100:0.1 to 50:50:0.1 *v*/*v*/*v*); Flow rate: 0.3 mL/min		
Viequeamide A (**90**)	Marine button cyanobacterium *Rivularia* sp.	l-Val, l-Thr, *N-*Me-l-Val, l-Pro	Marfey’s method (FDLA) combined with HPLC Conditions not described	Highly toxic to H460 (human lung cancer) cells	[99]
Ngercheumicin F–I (**91**–**94**)	Photobacterium related to *P. halotolerans*	l-Ser, l-*allo-*Thr, d-Ser, d-Thr, l-Leu, d-Leu	Marfey’s method (FDLA) combined with HPLC Dionex RSLC Ultimate 300 with a diode array detector Kinetex C_18_ column, 2.6 µm at 60 °C (2.1 × 150 mm) ACN:H_2_O:TFA (0:100:0.1 to 50:50:0.1 *v*/*v*/*v*) Flow rate: 0.8 mL/min	**91**–**93**: *rnaIII inhibiting* activities	[100]

aa—Amino acid; FDAA—1-Fluoro-2-4-dinitrophenyl-5-l-alanine amide; LC—Liquid Chromatography; MS—Mass Spectrometry; HPLC—High Performance Liquid Chromatography; MP—Mobile Phase; TEAP—Triethylammonium phosphate; ACN—Acetonitrile; TFA—Trifluoracetic acid; MeOH—Methanol; TEA—Triethylamine; IPA—Isopropyl alcohol; FDLA—1-Fluoro-2-4-dinitrophenyl-5-d,l-leucine amide; NaOAc—Sodium acetate; NH_4_OAc—Ammonium acetate.

**Table 3:** Lipopeptides from marine cyanobacteria.

Peptide	Source	aa Composition	Chromatographic Conditions	Biological Activities	Ref.
Antillatoxin B (**95**)	Cyanobacterium *Lyngbya majuscula*	*N*-Me-l-Hph	Marfey’s method (FDAA) combined with HPLC Waters Nova-Pak C_18_ (3.9 × 150 mm), MP: 10 to 50% ACN in H_2_O with 0.05% TFA, UV detection at 340 nm	Sodium channel-activating and ichthyotoxic activities	[101]
Lobocyclami-des A–C (**96**–**98**)	Cyanobacterium *Lyngbya confervoides*	**96**: *S*-Ile, *S-allo*-Ile, *S*-Leu, *R-*β-Aoa, *S*-Ser, *R*-Tyr, *S*-Hse, *R*-Hpr	Ligand Exchange Type CSP Chirex 3126 (d)-penicillamine column; MP: 2 mM aq CuSO_4_/ACN (1:99, 95:5 or 86:14 *v*/*v*); Flow rate: 1.15–1.20 mL/min, UV detection at 254 nm	Antifungal activity against a panel of *Candida* sp.	[102]
		**97**: *S*-Ala, *S*-Thr, *N*-Me-*S*-Ile, *R*-Aoa, *R*-Ada, 2*R*,3*R-*4-OH-Hth, 2*R*,3*S-*3-OH-Leu, *trans-*3-OH-Pro	Marfey’s method (FDAA) combined with HPLCC_18_ column (4.8 × 250 mm); MP: ACN: 0.1% aq. TFA buffer (pH 3) (1:9 to 1:1 *v*/*v*) Flow rate: 1.0 mL/min; UV detection at 340 nm		

aa—Amino acid; FDAA—1-Fluoro-2-4-dinitrophenyl-5-l-alanine amide; HPLC—High Performance Liquid Chromatography; MP—Mobile Phase; ACN—Acetonitrile; TFA—Trifluoracetic acid.

**Table 4:** Cyclic peptides from marine-derived fungi.

Peptide	Source	aa Composition	Chromatographic Conditions	Biological Activities	Ref.
Cyclo-(l-leucyl-*trans*-4-hydroxyl-l-prolyl-d-leucyl-*trans*-4-hydroxy-l-proline) (**99**)	Marine mangrove-derived fungi *Phomopsis* sp. K38 and *Alternaria* sp. E33	4-OH-l-Pro, d-Leu, l-Leu	Marfey’s method (FDAA) combined with LC/MS Alltima C_18_ column, 5 μm; (4.6 × 250 mm) MP: MeOH:H_2_O:HCOOH (60:40:0.05 to 10:90:0.05 *v*/*v*/*v*); Flow rate: 0.6 mL/min	Inhibition against four crop-threatening fungi	[105]
Scytalidamides A (**100**) and B (**101**)	Marine Fungus of the genus *Scytalidium*	l-Phe, *N-*Me-l-Phe, l-Leu, *N-*Me-l-Leu, l-Pro, 3-Me-l-Pro	Marfey’s method (FDLA) combined with HPLC Agilent Hypersil ODS column, 5 μm (4.6 × 100 mm); MP: ACN 25 to 65%; Flow rate: 0.7 mL/min	Cytotoxicity against HCT-116 and NCI 60 cell lines	[106]
Clonostachysins A (**102**) and B (**103**)	Marine sponge-derived fungus *Clonostachys rogersoniana* strain HJK9	*N*-Me-l-Ile, *N*-Me-l-Leu, l-Pro, l-Gly, *N*-Me-l-Tyr, *N*-Me-l-Ala **102**: *N*-Me-l-Val; **103**: *N*-Me-l-Ile	Marfey’s method (FDLA) combined with LC-ESI MS/MS; Conditions not described	Inhibitory effect on dinoflagellate *Prorocentrum micans*	[107]
Asperterrestide A (**104**)	Marine-derived fungus *Aspergillus terreus* SCSGAF0162	d-Ala	Marfey’s method (FDAA) combined with HPLC Alltima C_18_ column, 5 μm (4.6 × 250 mm); MP: ACN:H_2_O:TFA (15:85:0.1 to 90:10:0.1 *v*/*v*/*v*); Flow rate: 0.5 mL/min; UV detection at 254 nm	Cytotoxicity against U937 and MOLT4 human carcinoma cell lines and inhibitory effects on influenza virus	[108]
			Ligand Exchange Type CSP; MCI GELCRS 10 W (4.6 × 50 mm); MP: 2 mM CuSO_4_:H_2_O solution Flow rate: 1.0 mL/min; UV detection at 254 nm		
Sclerotides A (**105**) and B (**106**)	Marine-derived fungus, *Aspergillu*s *sclerotiorum* PT06-1	l-Thr, l-Ala, d-Phe, d-Ser	Crown Ether CSP; Crownpak CR (+); MP: aq HClO_4_ pH 2.0; Flow rate: 0.4 mL/min; UV detection at 200 nm	**105** and **106**: Antifungal activity **106**: Cytotoxicity and antibacterial activity	[109]
Cordyheptapeptides C–E (**107**–**109**)	Marine-derived fungus *Acremonium persicinum* SCSIO 115	*N*-Me-l-Tyr, l-Phe, l-Pro, l-Leu **107**–**109**: *N*-Me-d-Phe, l-Val **109**: *N*-Me-l-Gly, *N*-Me-d-Tyr, l-*allo*-Ile	Crown Ether Chiral CSP; Crownpak CR (+) MP: 2.0 mM CuSO_4_:ACN (95:5 *v*/*v*) Flow rate: 1.0 mL/min; UV detection at 254 nm	**107** and **109**: Cytotoxicity against SF-268, MCF-7, and NCI-460 tumor cell lines	[110]
Similanamide (**110**)	Marine sponge-associated fungus *Aspergillus similanensis* KUFA 0013	l-Ala, d-Leu, l-Val, *N*-Me-l-Leu, d-pipecolic acid	Macrocyclic Antibiotic Type CSP; Chirobiotic T, 5 μm (4.6 × 150 mm); MP: MeOH:H_2_O:CH_3_COOH (70:30:0.02 *v*/*v*/*v*); Flow rate: 1.0 mL/min; UV detection at 210 nm	Cytotoxicity against MCF-7, NCI-H460 and A373 tumor cell lines	[111]
Sartoryglabramide A (**111**) and B (**112**)	Marine sponge-associated fungus *Neosartorya glabra* KUFA 0702	l-Phe, l-Pro **112**: l-Trp	Macrocyclic Antibiotic Type CSP; Chirobiotic T, 5 μm (4.6 × 150 mm); MP: MeOH:H_2_O (80:20 *v*/*v*) Flow rate: 1.0 mL/min; UV detection at 210 nm	Neither antibacterial nor antifungal activity	[112]

aa—Amino acid; FDAA—1-Fluoro-2-4-dinitrophenyl-5-l-alanine amide; HPLC—High Performance Liquid Chromatography; MP—Mobile Phase; ACN—Acetonitrile; TFA—Trifluoracetic acid; MeOH—Methanol; FDLA—1-fluoro-2-4-dinitrophenyl-5-d,l-leucine amide.

**Table 5:** Cyclic depsipeptides from marine-derived fungi.

Peptide	Source	aa Composition	Chromatographic Conditions	Biological Activities	Ref.
Exumolides A (**113**) and B (**114**)	Fungus of the genus *Scytalidium* sp.	l-Pro, l-Phe, *N*-Me-l-Leu	Marfey’s method (FDAA) combined with HPLC Hewlett Packard 1090 Diode Array, 5 µm (10 × 250 mm); MP: 10–50% aq ACN (0.1% TFA) Flow rate: 1.0 mL/min; UV detection at 340 nm	Antimicroalgal activity against unicellular chlorophyte *Dunaliella* sp	[113]
Guangomide A (**115**)	Sponge-derived fungus	*N*-Me-d-Phe	Marfey’s method (FDAA) combined with HPLC Alltech Altima C_18_ column, 5 µm (10 × 250 mm) MP: ACN:H_2_O (4:1 to 1:1 *v*/*v*); Flow rate: 1.0 mL/min; UV detection at 340 nm	Antibacterial activity against *Staphylococcus epidermidis* and *Enterococcus durans*	[114]
Destruxin E chlorohydrin (**116**) and pseudodestruxin C (**117**)	Marine-derived fungus *Beauveria felina*	*N*-Me-l-Val **116**: *N*-Me-l-Ala, l-Ile **117**: l-Phe	Marfey’s method (FDAA) combined with HPLC C_18_ column, 5 µm (4.6 × 250 mm); MP: 10–20% ACN in 0.1 M NH_4_OAc (pH = 5) Flow rate: 1.0 mL/min; UV detection at 340 nm	Cytotoxicity in NCI’s 60 cell line panel	[115]
Zygosporamide (**118**)	Marine-derived fungus *Zygosporium masonii*	l-Phe, l-Leu, d-Leu	Marfey’s method (FDAA) combined with HPLC C_18_, Agilent column, 5 µm (4.6 × 250 mm) MP: 10–50% ACN (0.1% TFA) Flow rate: 1.0 mL/min; UV detection at 340 nm	Cytotoxicity in RXF 393 and SF-268 cancer cell lines	[116]
Petriellin A (**119**)	Coprophilous fungus *Petriella sordida*	*N*-Me-l-Ile, *N*-Me-l-Thr d-Phenyllactate	Marfey’s method (FDAA) combined with HPLC C_18_ column (4.6 × 250 mm); Conditions not described; UV detection at 260 nm	Antifungal activity	[117]
Alternaramide (**120**)	Marine derived fungus *Alternaria* sp. SF-5016	l-Pro, d-Phe	Marfey’s method (FDAA) combined with HPLC Capcell Pak C_18_ column; MP: 30–60% ACN in H_2_O (0.1% HCOOH); Flow rate: 1.0 mL/min	Antibacterial activity against *Bacillus subtilis* and *Staphylococcus aureus*	[118]
Petrosifungins A (**121**) and B (**122**)	*Penicillum brevicompac-tum*	l-Val, l-Pro, l-Thr, l-pipecolinic acids	Marfey’s method (FDAA) combined with HPLC C_18_ column, Waters, 5 µm (2.1 × 150 mm); MP: H_2_O or ACN (0.05% TFA); Flow rate: 1.0 mL/min	Not described	[119]
Oryzamides A–E (**123**–**127**)	Sponge-Derived fungus *Nigrospora oryzae* PF18	l-Ala, d-Leu, l-Val **123**: l-Leu; **124**: l-Tyr **125** and **126**: l-Met; **127**: l-Phe	Marfey’s method (FDLA) combined with UHPLC Acquity UHPLC BEH column, 1.7 µm (2.1 × 250 mm); MP: 10–100% ACN in H_2_O with 0.1% HCOOH; Flow rate: 0.5 mL/min; UV detection at 360 nm	No cytotoxicity, antibacterial, antiparasitic, and NF-*k*B activities	[122]
Spicellamide A (**128**) and B (**129**)	Marine-derived fungus *Spicellum roseum*	*N*-Me-d-Phe, *N*-Me-l-Ala, l-Ala	Marfey’s method (FDAA) combined with HPLC C_18_ column; Macherey-Nagel Nucleodur 100, 5 µm (2.0 × 125 mm); MP: MeOH:H_2_O (10:90 *v*/*v* to 100% MeOH) or 100% MeOH with NH_4_Ac, 2 mmol	**129**: Cytotoxicity	[123]
		l-2-hydroxyisocaproic acid	Ligand Exchange Type CSP; Phenomenex Chirex 3126 *N*,*S*-dioctyl-(d)-penicillamine (4.6 × 50 mm) MP: 2 mM CuSO_4_ in ACN:H_2_O (15:85 *v*/*v*) Flow rate: 1.0 mL/min; UV detection at 254 nm		
Depsipeptides 1962A (**130**) and 1962B (**131**)	Endophytic fungus *Kandelia candel*	l-Tyr, l-Val, d-Leu, (*S*)-*O*-Leu	Crown Ether CSP; Crownpak CR (+) column (0.4 × 150 mm), MP: 2 mM CuSO_4_ aq. solutions Flow rate: 0.5 mL/min; UV detection at 200 nm	**131**: Activity against MCF-7 tumor cell line	[124]

aa—Amino acid; FDAA—1-Fluoro-2-4-dinitrophenyl-5-l-alanine amide; HPLC—High Performance Liquid Chromatography; MP—Mobile Phase; ACN—Acetonitrile; TFA—Trifluoracetic acid; MeOH—Methanol; TEA—Triethylamine; FDLA—1-Fluoro-2-4-dinitrophenyl-5-d,l-leucine amide; NaOAc—Sodium acetate; NH_4_OAc—Ammonium acetate.

**Table 6:** Cyclic peptides from marine sponges.

Peptide	Source	aa Composition	Chromatographic Conditions	Biological Activities	Ref.
Haliclamide (**132**)	Vanuatu marine sponge *Haliclona* sp.	*N-*Me-l-Phe	Marfey’s method (FDAA) combined with HPLC Vydac C18; MP: ACN in H_2_O with 0.1% TFA (9:1 to 1:1 *v*/*v*); UV detection at 340 nm	Cytotoxicity against NSCLC-N6 carcinoma cell line	[129]
Microsclerodermins J (**133**) and K (**134**)	Deep water sponge *Microscleroderma herdmani*	l-Ile, l-Thr **133**: l-Phe, l-Gly **134**: l-Val, l-Ala	Marfey’s method (FDAA) combined with HPLC C_18_ column, 5 µm (4.6 × 150 mm) Flow rate: 1.0 mL/min; UV detection at 340 nm	Activity against opportunistic pathogenenic fungi	[130]
Euryjanicins E–G (**135**–**137**)	The Caribbean Sponge *Prosuberites laughlini*	l-Pro, l-Ile, l-Phe **135**: l-Asp	Marfey’s method (FDAA) combined with HPLC C_18_ column, 5 µm (4.6 × 150 mm) Flow rate: 1.0 mL/min; UV detection at 340 nm	No significant activity cytotoxicity against the National Cancer Institute 60 tumor cell line panel	[131]
Chujamide A (**138**)	Marine sponge *Suberites waedoensis*	l-Pro, l-Tyr, l-Cys, l-Leu, l-Phe l-Ile (*S*)	Marfey’s method (FDAA) combined with HPLC ESI-LC/MS YMC ODS-A column, 5 µm (4.6 × 250 mm) MP: H_2_O:ACN (80:20 to 30:70 *v*/*v*) Flow rate: 0.7 mL/min; UV detection at 360 nm	Weak cytotoxicity against A549 and K562 cell lines	[132]
Kapakahines A–D (**139**–**142**)	Marine Sponge *Cribrochalina olemda*	l-Val, l-Ile, l-Leu, l-Trp, l-Phe**,** l-Ala, l-Pro, l-Try	Marfey’s method (FDAA) combined with HPLC Cosmosil C_18_-MS column, 5 µm (4.6 × 250 mm) MP 37.5% ACN in 0.05% TFA or 20% or 38% ACN in 50 mM NH_4_OAc	**139**–**141**: Cytotoxicity against P388 cell line **139**: Inhibition against protein phosphatase	[133]
Koshikamide B (**143**)	Marine sponge *Theonella* sp.	d-Phe, l-Thr, *N*-Me-l-Val, *N*-Me-l-Asn, *N*-Me-l-Leu	Marfey’s method (FDAA) combined with HPLC ODS HPLC (10 × 250 mm); MP: ACN:H_2_O:TFA (25:75:0.05 to 55:45:0.05 *v*/*v*/*v*); Flow rate: 1.0 mL/min; UV detection at 340 nm	Cytotoxicity against P388 and HCT-116 tumor cell lines	[134]
Perthamides C (**144**) and D (**145**)	Solomon Lithistid sponge *Theonella swinhoei*	l-Asp, l-ThrOMe, (2*R*,3*S*)-βOHAsp, l-Phe	Marfey’s method (FDAA) combined with HPLC/MS Proteo C_18_ column (1.8 × 25 mm) MP: 10–50% aq ACN with 5% HCOOH and 0.05% TFA Flow rate: 0.15 mL/min	Anti-inflammatory activity	[135]
Perthamides E (**146**) and F (**147**)	Polar extracts of the sponge *Theonella swinhoei*	**146**: l-ThrOMe **147**: l-Phe	Marfey’s method (FDAA) combined with HPLC Proteo C_18_ column, (1.8 × 25 mm); MP: 10–50% aq ACN with 5% HCOOH and 0.05% TFA Flow rate: 0.15 mL/min	**147**: IL-8 release inhibition	[136]
Stylisins 1 (**148**) and 2 (**149**)	Jamaican sponge *Stylissa caribica*	l-Pro, l-Tyr, l-Ile **148**: l-Leu, l-Phe	Marfey’s method (FDAA) combined with HPLC HPLC water Nova Pack column (3.9 × 150 mm) MP: TEAP buffer (pH 3.0 ± 0.02):ACN (90 to 60% TEAP) UV detection at 340 nm	No antimicrobial, antimalarial, anticancer, anti-HIV-1, anti-Mtb and anti-inflammatory activities	[137]
Carteritins A (**150**) and B (**151**)	Marine sponge *Stylissa carteri*	**150**: l-Pro, l-Phe, l-Ile, l-Pro (*trans*), l-Pro (*cis*), l-Glu, l-Tyr; **151**: l-Pro (*trans*), l-Leu, l-Tyr, l-Pro(*cis*)	Marfey’s method (FDAA) combined with HPLC Cosmosil C_18_ MS (4.6 × 250 mm); MP: H_2_O:TFA (100:0.1) to ACN:H_2_O:TFA (50:50:0.1 *v*/*v*/*v*); Flow rate: 1.0 mL/min; UV detection at 340 nm	**150**: Cytotoxicity against HeLa, HCT116, and RAW264 cells	[139]
Stylissatins B–D (**152**–**154**)	Marine sponge *Stylissa massa*	l-Pro, l-Phe, l-Leu **152**: l-His **153**–**154**: l-Asp, l-Val	Marfey’s method (FDAA) combined with HPLC Thermo BDS Hypersil C_18_ column, 5 μm (4.6 × 150 mm); MP: 30–70% MeOH:H_2_O (H_3_PO_4_) Flow rate: 1.0 mL/min; UV detection at 340 nm	**152**: Inhibitory effects against a panel of human tumor cell lines including HCT-116, HepG2, BGC-823, NCI-H1650, A2780, and MCF7	[138]
Callyaerin G (**155**)	Indonesian sponge *Callyspongia aerizusa*	l-Pro, l-Leu, l-Phe, l-FGly	Marfey’s method (FDAA) combined with HPLC/MS; Conditions not described	Cytotoxicity against L5178Y, Hela, and PC12	[140]
Reniochalistatins A–E (**156**–**160**)	Marine sponge *Reniochalina stalagmitis*	l-Pro, l-Phe, l-Val, l-Leu, l-Ile, l-Tyr	Ligand Exchange Type CSP; MCI GELCRS 10 W (4.6 × 50 mm); MP: 2 mM CuSO_4_:H_2_O solution Flow rate: 1.0 mL/min; UV detection at 254 nm	**160**: Cytotoxicity against RPMI-8226, MGC-803, HL-60, HepG2, and HeLa	[141]
		**156**: l-Asn **160**: l-Trp	Marfey’s method (FDAA) combined with HPLC YMC-Park Pro C_18_, 5 µm (4.6 × 250 mm) MP: 2 mM CuSO_4_:H_2_O solution Flow rate: 1.0 mL/min; UV detection at 254 nm		
Phakellistatins 15–18 (**161**–**164**)	South china sea sponge *Phakellia fusca*	l-Pro **161**: l-Trp, l-Ile, l-Leu, l-Thr; **162**: l-Phe, l-Asp, l-Ser, l-Arg, l-Ala, l-Val, l-Thr, l-Tyr; **163**: l-Trp, l-Val, l-Leu, l-Ile; **164**: l-Tyr, l-Ile, l-Phe	Ligand-exchange type CSP; Chirex 3126 (d)-penicillamine column (4.6 × 150 mm) MP: aq 2 mM CuSO_4_:MeOH (85:15 to 70:30 *v*/*v*) or aq 1 mM/0.5 mM CuSO_4_; Flow rate: 0.5 or 1.0 mL/min	**161**: Cytotoxicity against P388 cancer cell line **162**: Cytotoxicity against P388 and BEL-7402 cancer cell lines	[142]

aa—Amino acid; FDAA—1-Fluoro-2-4-dinitrophenyl-5-l-alanine amide; LC—Liquid Chromatography; MS—Mass spectrometry; HPLC—High Performance Liquid Chromatography; MP—Mobile Phase; TEAP—Triethylammonium phosphate; ACN—Acetonitrile; TFA—Trifluoracetic acid; MeOH—Methanol; TEA—Triethylamine; IPA—Isopropyl alcohol; NaOAc—Sodium acetate; NH_4_OAc—Ammonium acetate.

**Table 7:** Cyclic depsipeptides from marine sponges.

Peptide	Source	aa Composition	Chromatographic conditions	Biological activities	Ref.
Callipeltins B (**165**) and C (**166**)	*Callipelta* sp.	l-Ala, d-Arg, l-Thr, *N*-Me-l-Ala, l-Leu	Marfey’s method (FDAA) combined with HPLC; Column not described; MP: TEAP (50 nM, pH 3.0):ACN 90–50% TEAP Flow rate: 2.0 mL/min; UV detection at 340 nm	Cytotoxicity **166**: Growth inhibitory activity against *Candida albicans*	[143]
Halipeptiins A (**167**) and B (**168**)	*Haliclona* species	l-Ala	Marfey’s method (FDAA) combined with HPLC Vydac C_18_ column; MP: H_2_O (0.1% TFA):ACN (0:1 to 1:1 *v*/*v*) UV detection at 340 nm	**168**: Anti-inflammatory activity	[144]
Phoriospongin A (**169**) and B (**170**)	*Phoriospongia* sp. and *Callyspongia bilamellata*	d-Asp, d-*allo-*Thr, d-Ala, l-Phe, d-Leu, d-*nor*-Val, *N-*Me-d-*nor*-Val **170**: *N-*Me-l-Leu	Marfey’s method (FDAA) combined with HPLC C_18_ column, 5 µm (4.6 × 250 mm) Flow rate: 1.0 mL/min; UV detection at 340 nm	Nematocidal activity against the parasite *Haemonchus contortus*	[145]
Mirabamides A–D (**171**–**174**)	*Siliquarias-pongia mirabilis*	*N*-Me-l-Thr, l-Thr, l-Ala, d-3-OMeAla, (2*R*,3*R*)-3-OH-Leu (3*S*,4*R*)-diMe-l-Glu, (2*S*,3*R*)-diaminobutanoic acid; **174**: l-HPr	Marfey’s method (FDAA) combined with HPLC Phenomenex Jupiter Proteo C_12_ column, 4 µm (4.6 × 150 mm) MP: 25–70% ACN; Flow rate: 0.5 mL/min	**171**: Anti-HIV activity **173** and **174**: Antibacterial activity **171**–**173**: Antifungal activity	[146]
Neamphamides B (**175**), C (**176**) and D (**177**)	*Neamphius huxleyi*	d-Arg, l-Asn, l-Hpr, l-Leu, d-*allo*-Thr **175** and **177**: *N*-Me-l-Gln **176**: *N*-Me-l-Glu	Marfey’s method (FDAA) combined with HPLC Phenomenex Luna Column C_18_, 3 µm (2.0 × 150 mm) MP: H_2_O:ACN:HCOOH (100:0:0.1 to 0:100:0.1 *v*/*v*/*v*) UV detection at 340 nm	Growth inhibition of human cell lines: A549, HeLa, LNCaP, PC3, and NFF	[147]
Pipecolidepsins A (**178**) and B (**179**)	*Homophymia lamellosa*	d-Asp, l-Leu, d-Lys, d-*allo*-Thr, (3*S*,4*R*) diMe-l-Glu, (2*S*,3*S*)-EtO-Asp, *N*-Me-l-Glu, l-Pip	Marfey’s method (FDAA) combined with HPLC Symmetry C_18_, 5 µm (4.6 × 150 mm); MP: 20–50% ACN (0.04% TFA) in H_2_O (0.04% TFA); Flow rate: 0.8 mL/min	Cytotoxicity against three human tumor cell lines (A-549, HT-29, and MDA-MB-231)	[148]
Stellatolide A (**180**)	*Ecionemia acervus*	*N*-Me-l-Ala, l-Leu, *N*-Me-l-Gln, *N-*Me-d-Ser, d-*allo*-Thr	Marfey’s method (FDAA) combined with HPLC Hewlett-Packard Hypersil BDS-C_18_, 4 µm (4.0 × 100 mm); MP: H_2_O (0.1% TFA):ACN (90:10 to 50:50 *v*/*v*); Flow rate: 1.0 mL/min	In in vitro antiproliferative activity	[149]
Cyclolithistide A (**181**)	*Theonella swinhoei*	nor-*S*-Val, *S*-Phe, S-Gln, *N*-Me-*S*-Leu, *S*-Ala, *S*-Allo-*S*-Thr	Marfey’s method (FDAA) combined with HPLC ODS (4.6 × 250 mm); MP: 100% H_2_O Flow rate: 2.0 mL/min; UV detection at 210 nm	Antifungal activity against *Candida albicans* (ATCC 24433)	[150]
Nagahamide A (**182**)	*Theonella swinhoei*	l-Val, l-Ser, 3*S*-AHBA	Marfey’s method (FDAA) combined with HPLC ODS column (4.6 × 250 mm); Conditions not described	Antibacterial activity	[151]
Theopapuamides B (**183**) and C (**184**), Celebesides A–C (**185**–**187**)	*Siliquarias-pongia mirabilis*	**185**: l-βMeAsn	Marfey’s method (FDAA) combined with HPLC/MS Phenomenex Jupiter Proteo C_12_ column, 4 µm (4.6 × 150 mm) MP: 25–70% ACN with 0.01 M TFA; Flow rate: 0.5 mL/min	**185**: Inhibits HIV-1 Entry **183**–**185**: Cytotoxic to human colon tumor cell line (HCT-116) **183** and **185**: Antifungal activity against *Candida albicans*	[152]
			Ligand Exchange Type CSP Phenomenex column, Chirex Phase 3126 (D) (4.6 × 150 mm); MP: 1 mM CuSO_4_:ACN (95:5 *v*/*v*)Flow rate: 0.5 mL/min; UV detection at 254 nm		
Theopapuamide (**188**)	Lithistid sponge *Theonella swinhoei*	*N-*Me-l-Leu, d-Asp, l-Leu, *N-*Me-l-Glu	Ligand Exchange Type CSP Chirex Phase 3126 (D), 5 µm (4.6 × 250 mm); MP: IPA: 2 mM CuSO_4_ (5:95 *v*/*v*) Flow rate: 1.0 mL/min; UV detection at 254 nm	Cytotoxicity against CEM-TART and HCT-cell lines	[153]
		d-allo-Thr	Marfey’s method (FDAA) combined with HPLCPhenomenex C_18_, 5 µm (4.6 × 250 mm); MP: 10–50% ACN in H_2_O (0.05% TFA); Flow rate: 1.0 mL/min; UV detection at 340 nm		
Mutremdamide A (**189**) and Koshikamides C–H (**190**–**195**)	*Theonella swinhoei* and *Theonella cupola*	**189**: *N*-Me-l-Val; **190**: *N-*Me-l-Val, *N-*Me-l-Asn, l-Asn, *N-*Me-l-Leu, l-Pro, *N-*Me-allo-l-Ile, d-Phe	Marfey’s method (FDAA) combined with HPLC LC-MS analysis using a C_12_ column, 4 µm (4.6 × 250 mm); MP: ACN with 0.01% TFA; Flow rate: 0.5 mL/min	**189**–**195**: Anti-HIV-1 activity	[154]
		**191** and **192**: *N-*Me-*allo*-l-Ile, *N-*Me-l-Val; **192**–**194**: *N-*Me-*allo*-l-Ile, l-Ala 1, d-Ala2, l-Asn	Marfey’s method (FDAA) combined with HPLC LC-MS, C_18_ column, 4 µm (4.6 × 250 mm); MP: 20 mM buffer (AF):ACN (3:1 to 3:7 *v*/*v*); Flow rate: 0.5 mL/min		
		**195**: *N*-Me-allo-l-Ile	Chiral HPLC (column not described); MP: 1 mM CuSO_4_:ACN (95:5 *v*/*v*); Flow rate: 0.5 mL/min; UV detection at 254 nm		

aa—Amino acid; FDAA—1-Fluoro-2-4-dinitrophenyl-5-l-alanine amide; LC—Liquid Chromatography; MS—Mass spectrometry; HPLC—High Performance Liquid Chromatography; MP—Mobile Phase; TEAP—Triethylammonium phosphate; ACN—Acetonitrile; TFA—Trifluoracetic acid; MeOH—Methanol; TEA—Triethylamine; NaOAc—Sodium acetate; NH_4_OAc—Ammonium acetate.

**Table 8:** Lipopeptides from marine sponge.

Peptide	Source	aa Composition	Chromatographic Conditions	Biological Activity	Ref.
Sulfolipo-discamides A–C (**196**–**198**)	Sponge *Discoderma kiiensis*	l-Uda, l-Gly	Marfey’s method (FDAA) combined with HPLC Cosmosil C_18_-MSII column (4.6 × 250 mm); MP: 100 mM NaClO_4_ in 60% ACN Flow rate: 0.8 mL/min	**196**: Cytotoxicity against P388 cell line	[155]

aa—Amino acid; FDAA—1-Fluoro-2-4-dinitrophenyl-5-l-alanine amide; HPLC—High Performance Liquid Chromatography; MP—Mobile Phase; ACN—Acetonitrile.

**Table 9:** Cyclic peptides from marine invertebrates and algae.

Peptide	Source	aa Composition	Chromatographic Conditions	Biological Activities	Ref.
Didomolamides A (**199**) and B (**200**)	Ascidian *Didemnum molle*	l-Thr, l-Ala, l-Phe **200**: l-Tzl	Marfey’s method (FDAA) combined with HPLC; MP: 50 mM (TEAP) buffer pH 3: ACN (9:1 to 1:1 *v*/*v*); Flow rate: 1.0 mL/min; UV detection at 340 nm	Cytotoxicity against A549, HT29 MEL28 tumor cell lines	[157]
Mollamides B (**201**) and C (**202**)	Tunicate *Didemnum molle*	l-Thr, l-Ile, l-Pro **201**: l-Val, l-Phe **202**: l-Ser, l-Leu	Marfey’s method (FDAA) combined with HPLC; MP: 50 mM TEAP, pH 3.0: ACN (90:10 to 60:40 *v*/*v*) or 40 mM NH_4_OAc, 70% ACN, and 30% MeOH (98:2 to 66:34 *v*/*v*) Flow rate: 1.0 or 0.8 mL/min; UV detection at 340 nm	**201**: Activity against HIV, *Plasmodium falciparum*, *Lieshmania donovan*, and cytotoxicity against H460, MCF7, SF-268 cell lines	[158]
Antatollamides A (**203**) and B (**204**)	Ascidian *Didemnum-molle*	l-Ile, l-Phe, l-Val, l-Pro, d-Ala	Marfey’s method (FDLA) combined with HPLC/MSHypersil Gold C_18_ column, 1.9 µm (2.1 × 50 mm); MP: H_2_O 0.1%; HCOOH:ACN (85:15 to 55:45 *v*/*v*) Flow rate: 0.5 mL/min	**203**: Weak cytotoxicity against a chronic lymphocytic leukemia cell line	[159]
Sanguinamide A (**205**)	*Nudibranch Hexabranchs sanguineus*	l-Pro, l-Ile, l-Ala, l-Phe	Marfey’s method (FDLA) combined with HPLC Agilent Zorbax SB-Aq C_18_ column, 5 µm (4.6 × 250 mm) MP: 80% (H_2_O: 0.1% HCOOH), 20% (ACN)	Antifungal activity	[160]
Gamakamide E (**206**)	Oysters *Crassostrea giga*	l-Met(O), *N*-Me-l-Phe, l-Leu, d-Lys, l-Phe	Marfey’s method (FDLA) combined with HPLC Conditions not described	No growth inhibition abilities	[161]

aa—Amino acid; FDAA—1-Fluoro-2-4-dinitrophenyl-5-l-alanine amide; HPLC—High Performance Liquid Chromatography; MP—Mobile Phase; ACN—Acetonitrile; TEAP—Triethylammonium phosphate; FDLA—1-Fluoro-2-4-dinitrophenyl-5-d,l-leucine amide; TFA—Trifluoracetic acid; MeOH—Methanol; TEA—Triethylamine; NH_4_OAc—Ammonium acetate.

**Table 10:** Cyclic depsipeptides from marine invertebrates and algae.

Peptide	Source	aa Composition	Chromatographic Conditions	Biological Activities	Ref.
Kahalalides A–F (**207**–**212**)	Mollusk *Elysia rufescens*	**207**: d-Val-5; **208**: l-Val-1, d-Val-2, d-*allo*-Thr-1; **209**: l-Val-3, d-Val-4, l-Thr-2; **210**: d-Val-2, d-*allo*-Thr-1	Marfey’s method (FDLA) combined with HPLC COSMOSIL 5C_18_-AR MP: ACN:H_2_O:TFA (42:48:0.05 *v*/*v*/*v*) or ACN:H_2_O:50 mM NH_4_OAc (20:80:0.01 *v*/*v*/*v*)	**207**: Antimalarial activity **211**: Activity against RSV II virus	[162]
**Table 10**. *Cont.*Tamandarins A (**213**) and B (**214**)	Ascidian of the family Didemni-dae	**213**: *S*-Lac, l-Pro, *N*-Me-d-Leu, l-Thr, (3*S*,4*R*,5*S*)-Ist **214**: *S*-Lac, l-Pro, *N*-Me-d-Leu, l-Thr 3*S*,4*R*)-Nst	Marfey’s method (FDAA) combined with HPLC Hewlett-Packard ODS Hypersil 5 µm (4.6 × 200 mm); MP: 0.1% TFA in H_2_O or MeOH; Flow rate: 1.0 mL/min; UV detection at 340 nm	**213**: Cytotoxicity against various human cancer cell lines	[163]
KahalalidesP (**215**) and Q (**216**)	Green alga *Bryopsis* sp.	l-Asp, l-Val, d-Leu, l-Ser, l-Hyp, l-Pro, l-Lys	Marfey’s method (FDAA) combined with HPLC COSMOSIL 5C_18_-AR-II (4.6 × 250 mm); MP: 0.1 M NH_4_OAc pH 3 or 90% aq ACN	No antimicrobial and no hemolytic activities	[164]
Kahalalide O (**217**)	Mollusk *Elysia ornata* and green alga *Bryopsis* sp.	l-Ile, l-Thr, d-*allo*-Thr, d-Tyr, l-Val	Ligand Exchange Type CSP Chirex (D) Penicillamine Column (4.6 × 250 mm); MP: 1.9 mM CuSO_4_ in ACN:H_2_O (5:95) or 2.0 mM CuSO_4_ in H_2_O; UV detection at 254 nm	No growth inhibition of P-388, A549, HT29 and MEL28 cancer cell lines	[165]
		d-Trp	Marfey’s method (FDAA) combined with HPLC COSMOSIL 5C_18_-AR; MP: ACN:H_2_O:TFA (37.5:62.5:0.05 *v*/*v*/*v*); Flow rate: 1.0 mL/min UV detection at 254 nm		

aa—Amino acid; FDAA—1-Fluoro-2-4-dinitrophenyl-5-l-alanine amide; HPLC—High Performance Liquid Chromatography; MP—Mobile Phase; ACN—Acetonitrile; FDLA—1-fluoro-2-4-dinitrophenyl-5-d,l-leucine amide; TFA—Trifluoracetic acid; MeOH—Methanol; TEA—Triethylamine; NH_4_OAc—Ammonium acetate.

**Table 11:** Lipopeptides from marine invertebrates and algae.

Peptide	Source	aa Composition	Chromatographic Conditions	Biological Activities	Ref.
Eudistomides A (**218**) and B (**219**)	Ascidian *Eudistoma* sp.	l-Pro, l-Ala, l-Leu **219**: l-Cyp	Ligand Exchange Type CSP Phenomenex Chirex 3126 (D) (4.6 × 250 mm); MP: 2 mM CuSO_4_, 2 mM CuSO_4_:ACN (95:5 or 85:15 *v*/*v*); Flow rate: 1.0 mL/min UV detection at 254 nm	No activity reported	[166]
Mebamamides A (**220**) and B (**221**)	Green algae *Derbesia marina*	l-Leu, l-Pro, d-Ala, l-Thr, l-Val, d-Phe, d-Ser	Ligand Exchange Type CSP Diacel CHIRALPAK (MA+) (4.6 × 50 mm); MP: 2.0 mM CuSO_4_, Flow rate: 1.0 mL/min; UV detection at 254 nm	No growth inhibitory activity against HeLa and HL60 cell lines	[167]

aa—Amino acid; MP—Mobile Phase; ACN—Acetonitrile.

**Table 12:** Chiral HPLC analysis of the acidic hydrolysates of **110**, **111** and **112** by co-injection with amino acids standards.

	Retention Time (min)		Retention Time (min)
d-Trp (A)	5.20	d-pipecolic acid (B)	14.67
l-Trp (A)	4.51	Acidic hydrolysate of **110** (B)	6.59, 7.20, 8.09, 8.83, 9.67, 10.57, 14.69
l-Val (B)	6.60	Acidic hydrolysate of **110** + dl-Val (co-injection) (B)	6.61, 7.31, 8.30, 8.10, 8.84, 9.70, 10.50, 14.95
d-Val (B)	8.32	Acidic hydrolysate of **110** + dl-Ala (co-injection) (B)	6.59, 7.19, 8.04, 8.81, 9.37, 9.70, 10.50, 14.90
l-Ala (B)	7.16	Acidic hydrolysate of **110**+ dl-Leu (co-injection) (B)	6.60, 6.76, 7.26, 8.04, 8.83, 9.67, 10.54, 15.02
d-Ala (B)	9.36	Acidic hydrolysate of **110** + dl-pipecolic acid (co-injection) (B)	6.58, 7.20, 8.09, 8.64, 8.84, 9.77, 10.64, 14.64
l-Leu (B)	6.78	Acidic hydrolysate of **110** + *N*-Me-l-Leu (co-injection) (B)	6.59, 7.20, 8.09, 8.83, 9.67, 10.57, 14.69
d-Leu (B)	9.67	Acidic hydrolysate of **111** (A)	1.91, 2.55, 2.86, 3.49, 3.89, 6.79
*N*-Me-l-Leu (B)	8.09	Acidic hydrolysate of **111** + dl-Phe (co-injection) (A)	1.87, 2.50, 2.89, 3.68, 5.01, 6.82
l-Phe (A)	3.81	Acidic hydrolysate of **111** + dl-Pro (co-injection) (A)	1.96, 2.60, 2.96, 3.52, 3,92, 6.70, 21.09
d-Phe (A)	5.00	Acidic hydrolysate of **112** (A)	1.93, 3.07, 3.80, 4.29, 4.60, 6.62
l-Pro (A)	6.72	Acidic hydrolysate of **112** + dl-Phe (co-injection) (A)	1.90, 3.10, 3.78, 4.39, 5.04, 6.70
d-Pro (A)	20.10	Acidic hydrolysate of **112** + dl-Pro (co-injection) (A)	2.04, 3.02, 3.72, 4.30, 4.60, 6.66, 19.40
l-pipecolic acid (B)	8.68	Acidic hydrolysate of **112** + dl-Trp (co-injection) (A)	1.93, 2.99, 3.70, 4.29, 4.60, 5.07, 6.33

Column, Chirobiotic T; Mobile phase, MeOH:H_2_O (80:20 *v*/*v*) (A) or MeOH:H_2_O:acetic acid (70:30:0.02 *v*/*v*/*v*) (B); Flow rate, 1.0 mL/min (A) or 0.5 mL/min (B); UV detection, 210 nm.
